# A single molecule assay to probe monovalent and multivalent bonds between hyaluronan and its key leukocyte receptor CD44 under force

**DOI:** 10.1038/srep34176

**Published:** 2016-09-29

**Authors:** Fouzia Bano, Suneale Banerji, Mark Howarth, David G. Jackson, Ralf P. Richter

**Affiliations:** 1 CIC biomaGUNE, Paseo Miramon 182, 20009 Donostia-San Sebastian, Spain; 2 MRC Human Immunology Unit, Weatherall Institute of Molecular Medicine, University of Oxford, Oxford, OX39DS, UK; 3 Department of Biochemistry, University of Oxford, Oxford, OX13QU, UK; 4 Université Grenoble Alpes - CNRS, Laboratoire Interdisciplinaire de Physique (LIPhy), BP 87, 38402 Saint Martin d’Hères, France; 5 University of Leeds, School of Biomedical Sciences and School of Physics and Astronomy, Leeds, LS2 9JT, UK

## Abstract

Glycosaminoglycans (GAGs), a category of linear, anionic polysaccharides, are ubiquitous in the extracellular space, and important extrinsic regulators of cell function. Despite the recognized significance of mechanical stimuli in cellular communication, however, only few single molecule methods are currently available to study how monovalent and multivalent GAG·protein bonds respond to directed mechanical forces. Here, we have devised such a method, by combining purpose-designed surfaces that afford immobilization of GAGs and receptors at controlled nanoscale organizations with single molecule force spectroscopy (SMFS). We apply the method to study the interaction of the GAG polymer hyaluronan (HA) with CD44, its receptor in vascular endothelium. Individual bonds between HA and CD44 are remarkably resistant to rupture under force in comparison to their low binding affinity. Multiple bonds along a single HA chain rupture sequentially and independently under load. We also demonstrate how strong non-covalent bonds, which are versatile for controlled protein and GAG immobilization, can be effectively used as molecular anchors in SMFS. We thus establish a versatile method for analyzing the nanomechanics of GAG·protein interactions at the level of single GAG chains, which provides new molecular-level insight into the role of mechanical forces in the assembly and function of GAG-rich extracellular matrices.

Glycosaminoglycans (GAGs), a family of linear and anionic polysaccharides, are abundant in the extracellular space of vertebrates and act as vital regulators of the interactions between cells and their local environment. Simplest in structure among GAGs, hyaluronan (HA) is a regular polymer of disaccharides, composed of glucuronic acid and N-acetylglucosamine linked *via* alternating β-1,4 and β-1,3 glycosidic bonds, that can reach a contour length of several micrometers. The functional diversity of HA arises from its ability to bind various proteins, termed hyaladherins. These bind to the flexible HA chains and promote their self-assembly in hydrogel-like multimolecular complexes that frequently undergo further dynamic re-modelling. Such HA-rich matrices are of prime importance for regulating cell migration in key physiological processes such as inflammation^1^ and fertilization^2^, and in disease processes such as tumor metastasis^3^. HA also binds cell surface receptors, among which CD44 is structurally and biochemically the best characterized to date. In the vasculature, HA·CD44 interactions are known to mediate the recruitment of activated lymphocytes and neutrophils^4^,^5^ from the blood circulation, and have also been implicated in the recruitment of circulating stem cells^6^ and tumor cells^7^,^8^.

Cells migrating within the extracellular matrix are profoundly affected by physical forces and the mechanical properties of their environment. This realization has driven efforts to probe and understand how forces act on the molecular scale^9^. A prime example is the capture of activated leukocytes from the blood flow during inflammation, where mechanical forces are of vital importance in regulating adhesive interactions between PSGL-1 on the leukocyte surface and selectins on the blood vessel endothelium and the ensuing cell adhesion and rolling^10^,^11^. In this particular scenario, interactions between HA and CD44 also experience the shear stress of the blood flow^4^,^7^,^12^. More generally, given the structural role of GAG-protein interactions in the extracellular space, it is clear that these interactions are subjected to mechanical forces when matrix or tissues are deformed. To determine the biomechanical consequences of GAG-protein interactions, new methods are needed to probe the mechanical response of GAG-protein interactions at the nanoscale. On the most basic level, individual bonds need to be probed, and then, to better understand complexity in real biological systems, it must be determined how these elementary interactions are integrated into multivalent supramolecular systems. This is particularly important for GAGs because the polysaccharide chains can bind multiple proteins simultaneously.

Single molecule force spectroscopy (SMFS) is a powerful tool to study how forces affect molecular interactions, and to identify the underlying molecular mechanisms. In particular, atomic force microscopy (AFM) based SMFS is now well established for probing intra- and inter-molecular forces^13^,^14^,^15^. A few SMFS studies have probed GAG-protein interactions on the single bond level^16^,^17^,^18^,^19^, and similar assays have revealed specific and multivalent interactions between GAGs^20^, or GAG analogues in the case of lower organisms^21^, on complex proteoglycans. To date however, only one study has applied SMFS to probe multivalent GAG-protein interactions^17^. Furthermore, these were not analysed in detail, and the particular assay system allowed only limited control over the orientation of the immobilized protein. Seminal studies (reviewed in ref. 22) with DNA, another biological polymer, not only demonstrate the unique ability of SMFS approaches to de-construct the dynamic and hierarchical assembly of large supramolecular complexes such as chromatin but they also highlight the critical importance of controlling the immobilization of such complexes for successful measurements. One of the main challenges to obtaining physiologically meaningful data using SMFS is achieving a well defined spatial arrangement of the studied molecules in their native orientation, while at the same time permitting the controlled application of the necessary tensile forces.

Here, we have devised such a method for GAG·protein interactions, by combining purpose-designed surfaces that afford immobilization of GAGs and receptors in the form of controlled nano-scale organizations with AFM SMFS. More specifically, the tailored surface functionalization and characterization by quartz crystal microbalance (QCM-D) and spectroscopic ellipsometry (SE) has enabled the anchoring of receptors and HA to solid substrates at controlled orientation, grafting density and lateral mobility. The new assay system has allowed us to study *monovalent* interactions between the leukocyte receptor CD44 and its HA ligand at the single molecule level as well as *multivalent* interactions between individual HA chains and CD44-coated surfaces in well-defined spatial arrangements. These measurements reveal that CD44·HA bonds have a high tensile strength despite their low affinity, and that multiple bonds along an HA chain rupture independently under load. We also demonstrate that strong but non-covalent bonds such as those between biotin and streptavidin (SAv), or between polyhistidine and metal chelators, which are ideal tools for immobilization of biomolecules at well-defined orientation and densities, are sufficiently strong to be used as anchors in the force spectroscopy of GAG·protein interactions. Our new method should thus be widely applicable to the study of GAG·protein interactions.

## Results

In multivalent interactions, the (supra)molecular arrangement of the interaction partners can be critically important, and we therefore applied particular care in controlling the immobilization of both GAGs and proteins. Correct functionalization of surfaces with HA and its receptor CD44 was ascertained through quartz crystal microbalance (QCM-D) characterization.

### Immobilization of HA

**Figure 1:**
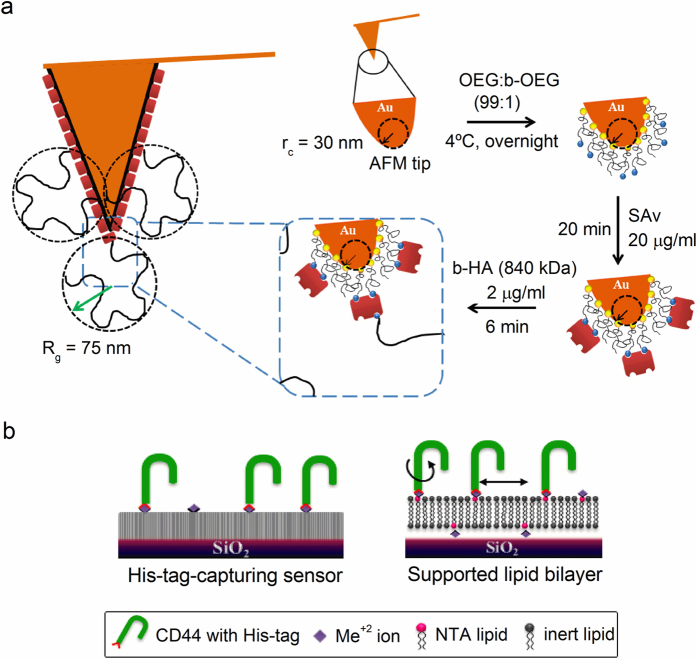
Surface functionalization with HA and CD44. (**a**) Representation (not to scale) of the protocol used to functionalize AFM probes with end-biotinylated HA (b-HA; 840 kDa) *via* monolayers of streptavidin (SAv) formed on a gold-supported mixed monolayer of thiolated oligo(ethylene glycol) with (b-OEG) and without (OEG) biotin. *R*_g_ is the radius of gyration of a single HA chain and *r*_c_ the radius of curvature of the apex of the AFM probe. (**b**) Representation of the architecture of surfaces (not to scale) displaying the cell-surface HA receptor CD44 either immobile (*i.e.*, His-tag-capturing layer; left) or laterally mobile (*i.e.*, SLB; right).

HA polymers of well-defined molecular mass (840 ± 60 kDa; contour length 2.10 ± 0.15 μm^23^) were immobilized by end-grafting through a biotin tag at the reducing end. This method has been described previously^24^,^25^ and was applied here to AFM probes with incubation conditions adjusted such that only one or at most a few HA molecules are able to contact the protein-covered surface (Fig. 1a and Supplementary Fig. S1). In our force spectroscopy assays, polymeric HA fulfils three distinct functions. First, it serves as a flexible linker to discriminate specific interactions from undesired non-specific interactions that may occur when the AFM tip is close to the surface^26^. Second, the HA polymer is long enough to accommodate several hundred HA-binding proteins simultaneously, and thus enables the study of both single bond and multivalent interactions between an individual HA chain and a protein-coated surface under load. The end-grafted HA chain transmits force to HA-receptor bonds in a well-defined and controlled way, thus facilitating quantitative data analysis.

### Optimization of receptor immobilization procedures by QCM-D

The immobilization strategies for CD44 were devised to provide stable and specific immobilization at controlled orientation and lateral mobility, and at tunable surface densities. Specifically, the CD44 construct consisted of the extracellular domain (ECD) and a C-terminal His_10_ tag for immobilization. CD44 was bound to either His-tag-capturing sensor surfaces *via* a Cu^2+^ chelate, or to supported lipid bilayers (SLBs) *via* a Ni^2+^-tris-nitrilotriacetic acid (NTA) chelate^27^, in a way that reproduces the native orientation of the HA binding domain on the plasma membrane (Fig. 1b).

**Figure 2:**
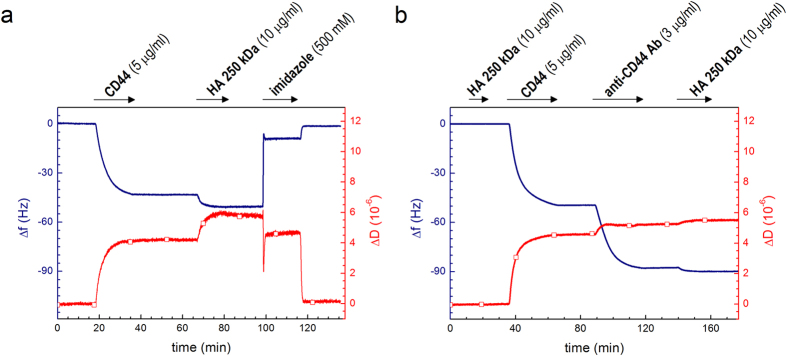
QCM-D immobilization assays for CD44 on His-tag-capturing sensors. QCM-D responses in (**a**) indicate formation of a stable and HA-binding CD44 monolayer, and demonstrate that CD44 is specifically immobilized through its polyhistidine tag (*i.e.*, it can be fully eluted in imidazole). Data in (**b**) demonstrate that HA binds through the authentic HA-binding site on CD44 (largely blocked with anti-CD44 Ab). The magnitude of the final shifts upon binding of HA polymer to CD44-coated surfaces were *∆f* = −7.4 ± 1.0 Hz and *∆D* = 1.7 ± 0.2 × 10^−6^.

QCM-D frequency and dissipation shifts (Δ*f* and Δ*D*, respectively) upon exposure of CD44 to His-tag-capturing sensor surfaces showed saturable binding (Fig. 2), the magnitudes of which were consistent with the formation of a protein monolayer. Indeed, the frequency shift close to saturation (*∆f* = −47 ± 4 Hz) corresponds to a film thickness of about 7 nm (see Methods), a value that is larger than the size of the HA binding domain alone (3.5 nm)^28^,^29^ and compatible with the estimated molecular mass (60 kDa) of the glycosylated CD44 domain (the exact dimensions of the full ECD are not known to our knowledge). CD44 remained stably bound throughout the buffer washing step, but was fully dissociated by elution with imidazole (Fig. 2a), confirming its immobilization solely *via* the polyhistidine tag. HA polymers (250 kDa) adsorbed readily and stably to the immobilized CD44 monolayers (Fig. 2a; ∆*f* = −7.4 ± 1.0 Hz), whereas no binding was observed to bare sensors and only residual binding (*∆f* = −2 Hz) persisted when the HA-binding site of CD44 was impeded with the function-blocking anti-CD44 monoclonal antibody BRIC235^30^ prior to HA incubation (Fig. 2b). The residual HA binding in this case most likely reflects incomplete blocking, *e.g.* due to spatial constraints of the large antibodies on the small receptor domains. These data clearly demonstrate that HA engages the authentic HA binding site on CD44. Given the low HA binding affinity for soluble CD44 as determined from previous surface plasmon resonance analyses (*K*_D_ = 10 to 100 μM)^31^, the stable binding we observed to immobilized receptor most likely arises from multivalent ligand interactions^30^.

Measurements of HA binding to CD44 immobilized on SLBs yielded similar results (Supplementary Fig. S2). Importantly, these SLBs retain the lateral mobility of CD44 in contrast to the conventional His-tag-capturing sensors in which the protein remains fixed^32^. In this way, the effect of receptor mobility on the interaction with HA can be independently assessed.

### Immobilization of proteins for AFM SMFS

The immobilization procedures for CD44 established by QCM-D were then adopted to prepare samples for AFM SMFS. The solution concentration of CD44 was varied to generate surfaces with desired protein coverage, where the protein surface density was estimated from spectroscopic ellipsometry (SE) measurements and where we exploited the fact that the protein binding rate is largely mass transport limited (Supplementary Fig. S3). In the following, we operationally define root-mean-square (rms) distances between receptors around 50 nm (comparable to the size of the HA coil, *R*_g_ ≈ 75 nm; Fig. 1) as ‘low receptor density’, and rms distances around 10 nm as ‘high receptor density’.

We stress that the correct orientation of the immobilized proteins is critical for their functionality. For example, when another HA-binding protein, the aggrecan G1 domain complex with cartilage link protein^33^ was immobilized through a randomly positioned tag, we detected only residual binding by QCM-D and AFM SMFS, revealing that only a very small fraction of the immobilized proteins engaged efficiently with HA (Supplementary Fig. S4).

### Single molecule force spectroscopy of individual HA·CD44 bonds

**Figure 3:**
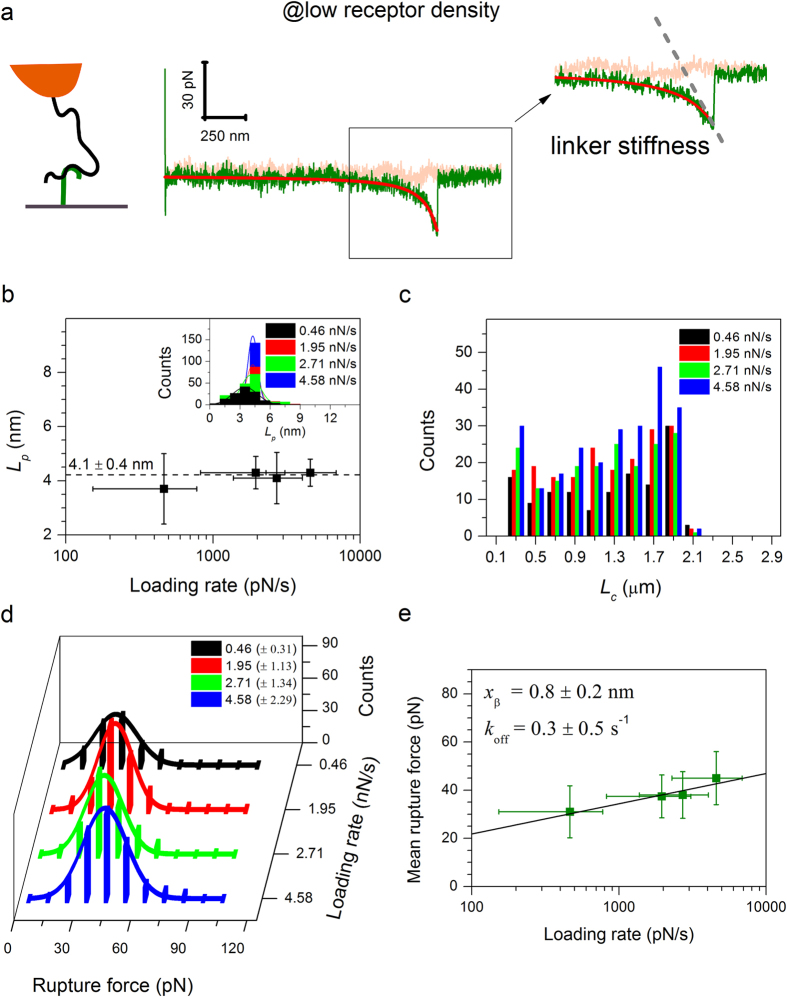
Analysis of HA·CD44 single-bond interactions. Representative force-separation curve (pink – approach, green – retract; retract velocity 1000 nm/s) recorded at low CD44 surface density (rms inter-CD44-distance ~50 nm) for a specific single unbinding event as schematically shown; the inset on the right shows an enlargement of the boxed portion of the force curve with an expanded separation axis. The red line represents a best-fit worm-like chain (WLC) model curve for HA stretching. The slope of this curve at the end of the extension curve (dashed line) corresponds to the linker stiffness and, together with the retraction speed, gives the instantaneous loading rate at the moment of bond rupture. (**b–e**) Results of the statistical analysis of single-rupture-event force curves, obtained at low CD44 density, with the WLC model. (**b**) Persistence length (*L*_p_) of HA as a function of instantaneous loading rate. The dashed line marks the mean *L*_p_ value, with mean ± s.d. indicated. The inset shows histograms of *L*_p_ for the studied instantaneous loading rates (as listed with color codes) with best-fit Gaussian curves. (**c**) Histograms of *L*_c_, *i.e.* the contour length of the HA chain from its anchor point to the CD44 binding locus, for the studied instantaneous loading rates (as listed with color codes). The largest measured *L*_c_ is comparable to the total contour length of the employed HA chains (2.1 μm), as expected. (**d**) Rupture force histograms for the different instantaneous loading rates (listed in the back panel as mean ± s. d.). The solid lines represent best-fit Gaussian curves. (**e**) Dynamic force spectra obtained from the data in (**d**); error bars represent s.d. The black line represents the best-fit Bell-Evans model curve with kinetic parameters indicated.

We first used force spectroscopy to characterize the forces associated with the unbinding of HA from a single CD44 molecule (Fig. 3 and Supplementary Fig. S5). Predominantly single rupture events were observed when the HA-bearing AFM probe was exposed to His-tag-capturing surfaces with low CD44 density (rms distance ~50 nm; Fig. 3a). Several observations confirm that these curves indeed represent the genuine interaction of a single HA chain with a single CD44 molecule. First, most (78%) of the force curves (*n* > 600) showed no rupture event, and only a minor fraction (9%) showed two or more distinct rupture events: a distribution that would be predicted for stochastic single-molecule interactions. Second, the HA stretching curves in every case overlapped when the distances were normalized according to predictions of the worm-like chain (WLC) model (Supplementary Fig. S5a)^34^. Third, no rupture events (in *n* = 200 force curves per condition) were observed when either CD44 or HA were omitted, indicating that the interactions are specific to these components (Supplementary Fig. S5b). Fourth, statistical analysis of force-separation curves with a single rupture event, acquired over a spectrum of retract velocities, using the WLC model revealed a velocity-independent persistence length (*L*_p_ = 4.1 ± 0.4 nm; Fig. 3b). The magnitude of *L*_p_ agrees with previously reported values obtained from single-molecule HA stretching experiments at comparable ionic strength and pH values (4.4 ± 1.2 nm)^35^. Fifth, the contour length *L*_c_ between the anchor point of HA and the CD44-binding locus varied broadly between measurements, and the maximum observed value matched the total contour length of the employed HA chains (2.1 μm) well (Fig. 3c). This confirms that a single HA chain is being stretched in our experiments and that CD44 can bind (with similar likelihood) at any position along that chain.

Histograms of the rupture forces revealed unimodal distributions for all retract velocities (Fig. 3d), suggesting that one specific bond is being probed. Indeed, the mean rupture force *F* depended linearly on the logarithm of the instantaneous loading rate *r* (Fig. 3e), as predicted by the Bell-Evans model^13^,^36^,^37^,^38^ for stochastic bond rupture under external load across a single energy barrier


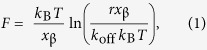


where *k*_B_*T* is the thermal energy, *x*_β_ is the width of the energy barrier, and *k*_off_ is the unbinding rate constant in the absence of external load. Fitting of the data with Eq. (1) (Fig. 3e, line) yielded *k*_off_ = 0.3 ± 0.5 s^−1^ and *x*_β_ = 0.8 ± 0.3 nm.

We note that the above-mentioned minor fraction of force curves showing more than one rupture event (9%) might originate from the binding of an HA chain to several distinct CD44 molecules. Given the large size of HA, it is though also possible that rebinding to the same receptor molecule occurs during the retract phase. The dominance of force curves with single binding events over multiple binding events indicates that re-binding is a rare phenomenon under the experimental conditions.

### Multivalent HA·CD44 interactions

**Figure 4:**
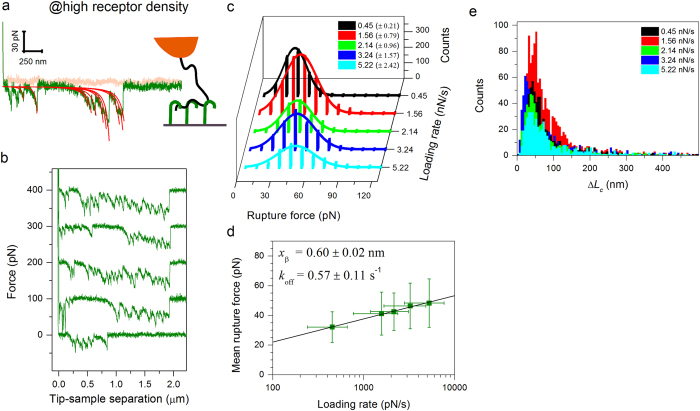
Analysis of HA·CD44 multivalent interactions. (**a**) Representative force-separation curve (pink – approach, green – retract; retract velocity 2000 nm/s) recorded at high CD44 surface density (rms inter-CD44-distance ~10 nm) for specific multiple unbinding events as schematically shown. The red lines represent best-fit WLC model curves (*L*_p_ = 4.1 nm fixed). (**b**) Five representative force-separation curves obtained under the same conditions; the curves are offset along the force axis for clarity. Each force curve is distinct, illustrating that bonds form stochastically at random positions along the HA chain. (**c–e**) Results of the statistical analysis of multiple-rupture-event force curves obtained at high CD44 density. (**c**) Rupture force histograms displayed analogous to Fig. 3d. (**d**) Dynamic force spectra displayed analogous to Fig. 3e. The similarity with the dynamic force spectra extracted from single-rupture-event curves (Fig. 3e) indicates that bonds rupture individually. (**e**) Histograms of HA loop length, equivalent to the contour length difference Δ*L*_c_ between successive rupture events.

Next, we used His-tag-capturing surfaces with high CD44 density (rms distance ~10 nm) to examine multivalent HA·receptor interactions. On these surfaces, force curves with a single rupture event were rare and multiple discrete unbinding peaks, typically around 10, were instead commonly observed (Fig. 4a,b).

The individual peaks in the force curves could be fitted closely (Fig. 4a) with a WLC model in which *L*_p_ was fixed to the previously established value of 4.1 nm. Detailed analysis over a spectrum of loading rates again revealed unimodal distributions of rupture forces (Fig. 4c), with distribution means and widths (Fig. 4d) that were fully consistent with the values obtained for interactions of an HA chain with a single CD44 molecule (Fig. 3e). The close match of the best-fit model curves with fixed *L*_p_ with the experimental data and the consistent distribution of rupture forces imply that all rupture events occur on the same HA chain, and that the individual bonds rupture independently from one another under load. The drastically enhanced number of rupture events at high CD44 surface density provided a larger dataset for statistical analysis (Fig. 4c) thus improving the accuracy of the determination of kinetic parameters through the Bell-Evans model (Fig. 4d): consolidated values were a barrier width *x*_β_ = 0.60 ± 0.02 nm and a dissociation rate constant *k*_off_ = 0.57 ± 0.11 s^−1^.

From the sample force curves in Fig. 4a,b, it can be readily appreciated that the spacing between subsequent rupture events in a given force curve is irregular, and that the location of rupture events across different force curves varies widely. This indicates that HA interconnects CD44 molecules through loops of varying size, a picture that is consistent with simple theoretical predictions for the adsorption of flexible polymers to surfaces^39^ and also with an earlier report on the thickness of films of HA polymers bound to CD44-coated surfaces^30^. The difference in contour length between successive rupture events provides a measure for the contour length of a loop, and the histogram (Fig. 4e) revealed a broad distribution of loop sizes: the loop size distribution had a maximum around 50 nm, but loops of several 100 nm contour length could also be observed.

### Role of lateral mobility in the rupture of HA·CD44 bonds

**Figure 5:**
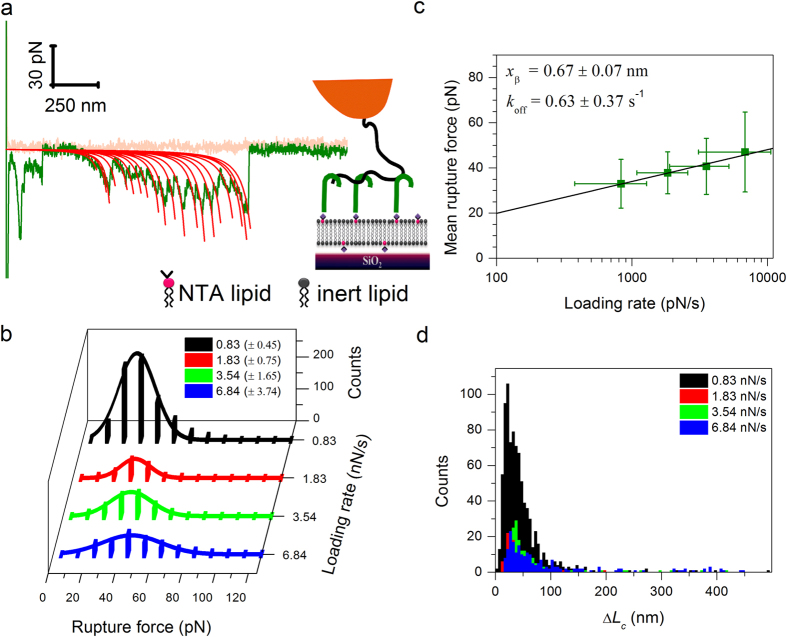
Effect of lateral mobility of CD44 on the interaction with HA. (**a**) Representative force-separation curve (approach/retract velocity 1000 nm/s) obtained between a HA-modified tip, and CD44 anchored to an SLB at high receptor density (rms inter-CD44-distance ~10 nm) as schematically shown (see also Supplementary Fig. S2). The force curve is qualitatively similar to those obtained for fully immobilized CD44 (*cf.* Fig. 4a,b). The red lines represent best-fit WLC model curves (*L*_p_ = 4.1 nm fixed). (**b–d**) Results of the statistical analysis of multiple-rupture-event force curves. (**b**) Rupture force histograms displayed analogous to Fig. 3d. (**c**) Dynamic force spectra displayed analogous to Fig. 3e. (**d**) Histograms of HA loop length, equivalent to the contour length difference Δ*L*_c_ between successive rupture events. These data are comparable to Fig. 4c–e, indicating that CD44 lateral mobility does not substantially affect the unbinding process.

The experiments thus far assessed the behavior of immobilized CD44. However, under normal physiological conditions, HA receptors may be mobile in the cell membrane. To test how lateral mobility affects the unbinding of multivalent HA-receptor interactions, we performed complementary measurements with CD44 attached to SLBs. As shown in Fig. 5, the force curves and the distribution of rupture forces were comparable to those obtained on His-tag-capturing sensors (see *e.g.* Fig. 4), *i.e.* the receptor’s lateral mobility does not substantially affect the unbinding process.

On the basis of all these findings, we conclude that individual HA·CD44 bonds are remarkably resistant to rupture under load considering their relatively low affinity, and that HA binds multivalently to a CD44-covered surface through the stochastic formation of loops, with bonds rupturing sequentially and independently under load. In particular, these results illustrate that our approach is well suited for the characterization of monovalent and multivalent interactions with a single platform under well-defined interaction conditions.

### Are CD44·HA bonds being probed?

The rapid, strong and specific interactions between biotin and SAv, and between polyhistidine and metal chelators, are ideal for the anchorage of proteins and glycans at controlled orientation and density. Moreover, supported lipid bilayers with functionalized lipids being retained in the membrane through hydrophobic interactions are a unique platform to produce well-defined surfaces with laterally mobile receptors. However, given that these interactions are non-covalent, there is a finite possibility that an anchor point yields upon application of a tensile force, and it is thus *a priori* not clear if the above-determined parameters characterize the genuine HA-receptor interaction. To resolve this question, we performed a set of control experiments with distinct anchors.

First, we replaced streptavidin as an anchor for HA by traptavidin (TAv), a streptavidin variant that binds more stably to biotin (Supplementary Fig. S6)^40^. The distributions of rupture forces with this modification were virtually identical compared to those obtained with SAv. Consequently, the kinetic parameters derived from the force spectra also agreed within experimental error (compare Supplementary Fig. S7c,d with Fig. 4c,d; Table 1). Moreover, single molecule force spectroscopy with biotin (attached to the AFM tip *via* a polyethylene glycol linker and a thiol-gold bond) and monolayers of SAv or TAv (prepared identically to the coatings used for HA attachment; Supplementary Figs S1 and S6) confirmed the enhanced mechanostability of TAv·biotin over SAv·biotin in our setup (Supplementary Fig. S8). Specifically, the mean rupture forces with TAv were increased by 10 to 20 pN compared to SAv within the range of loading rates tested (0.8 to 10 nN/s), and the derived kinetic parameters were comparable to those previously reported^40^.

Second, we replaced the His-tag-capturing surface by a SAv monolayer for the anchorage of CD44 (Supplementary Fig. S9a–c). Our CD44 construct featured a biotin tag side by side with the polyhistidine tag at the C-terminus, and thus enabled the effect of the anchors to be compared at virtually identical protein orientation on the surface. As for HA, the change in CD44 anchor had no substantial effect on the distribution of rupture forces and the derived kinetic parameters (compare Supplementary Fig. S9e,f with Fig. 4c,d; Table 1).

These control experiments demonstrate that the anchorages of HA *via* biotin and SAv, and of CD44 *via* polyhistidine and metal chelator, are strong enough and do not affect the force spectroscopy of HA·CD44 interactions appreciably. Moreover, the similar results obtained on SLBs (compare Fig. 5c,d with Fig. 4c,d; Table 1) imply that the anchorage of polyhistidine-binding lipids in the lipid bilayers is also strong enough. To explain this mechanistically, we performed in addition a theoretical analysis of rupture probabilities of bonds connected in series. We assumed that *n* bonds in a chain can rupture independently from each other, each according to the Bell-Evans model. Adopting the approach described by Neuert *et al*.^41^, the probability *φ*^(*i*)^ of bond *i* to rupture as a function of force *f* and loading rate *r* is defined by a system of *n* + 1 coupled ordinary differential equations


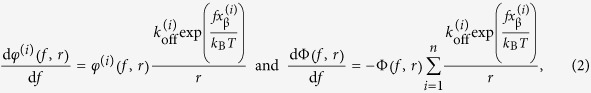


where 
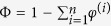
 is the probability of the *n*-bond chain to remain intact. The boundary conditions to solve these equations are defined by the *n*-bond chain being intact at the start of the measurement (Φ = 1 and 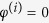 at *f* = 0).

To compare with our experiments, we considered for simplicity a system with two reversible bonds, representing CD44·HA and a SAv·biotin anchorage (thus effectively assuming the second anchorage to be irreversible). For SAv·biotin, we used the experimentally determined values for *k*_off_ and *x*_β_ (Supplementary Fig. S8f; Table 1) as input parameters. For CD44·HA, we used the values shown in Fig. 4d (and Table 1), as our control experiments had shown that these represent the individual CD44·HA interaction adequately even though they were obtained in a system with multiple bonds in series.

**Figure 6:**
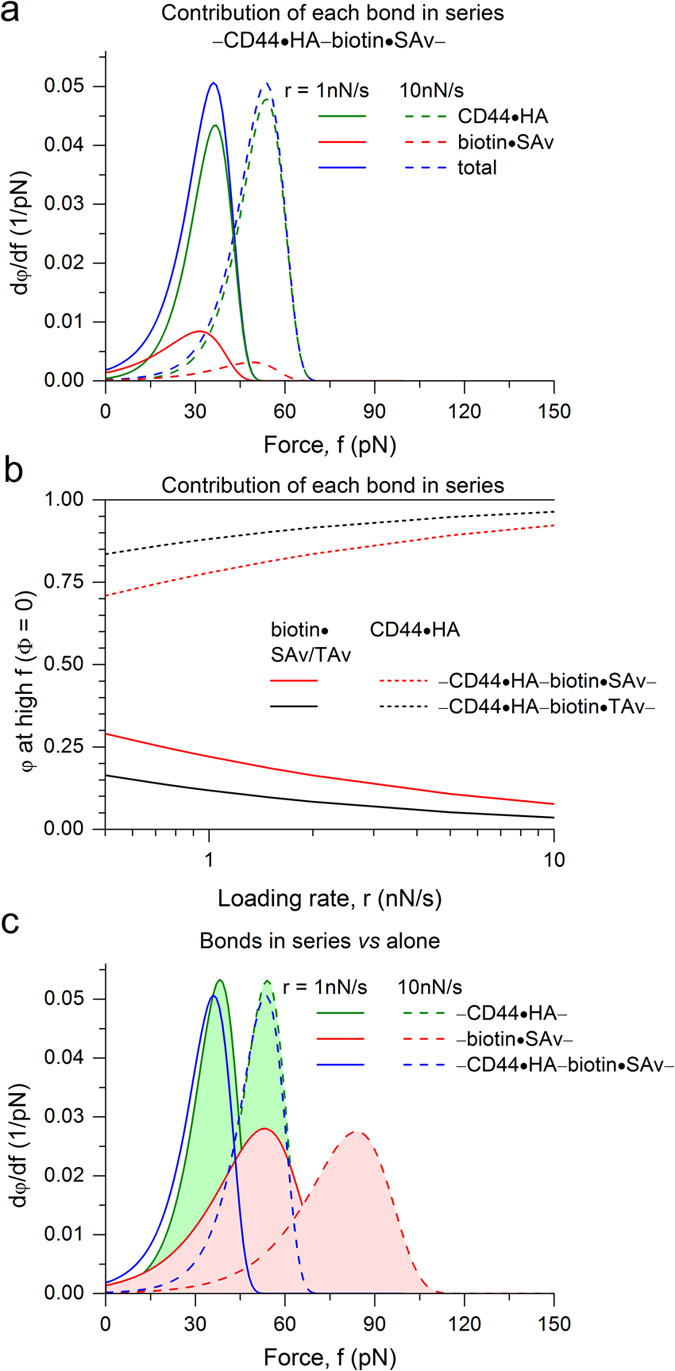
Theoretically predicted rupture probabilities for two reversible bonds in series. (**a**) Derivative of the rupture probability d*φ*/d*f vs.* force *f* for the scenario of CD44·HA in series with SAv·biotin at loading rates *r* = 1 nN/s (solid lines) and 10 nN/s (dashed lines); data for 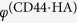 is shown in green, for 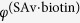 in red, and for the total rupture probability 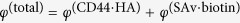 in blue. (**b**) Rupture probabilities *φ* in the limit of high forces (Φ = 0) *vs.* loading rate *r* for the scenarios of CD44·HA in series with SAv·biotin (red) or TAv·biotin (black); 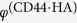 is shown as solid lines, 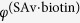 and 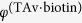 as dashed lines. The rupture of CD44·HA dominates over SAv·biotin over the entire range of loading rates, and the probability of the anchor to rupture is reduced by approximately two-fold upon replacing SAv·biotin by TAv·biotin. (**c**) Total rupture probabilities, displayed as d*φ*/d*f vs.* force *f*, for the scenario of CD44·HA in series with SAv·biotin (as shown in (**a**); blue lines) compared to CD44·HA alone (light green filling), and SAv·biotin alone (light red filling). The curves for CD44·HA alone are very similar to the curves for the two bonds in series, demonstrating that the occasional breakage of SAv·biotin, when in series with CD44·HA, does not affect the force spectra appreciably. See Table 1 and main text for the input data of the theoretical model.

Figure 6a shows the results of the numerical calculations in the form of the derivative dφ/d*f* of the rupture probability as a function of the force *f* at two selected loading rates (1 and 10 nN/s) that are close to the lowest and highest loading rates used experimentally, respectively. This plot reveals that the breakage of CD44·HA dominates over SAv·biotin, but also that the probability of SAv·biotin to break is sizeable.

Figure 6b shows the rupture probability *φ* of the individual bonds in the limit of high forces when all chains have effectively ruptured (Φ = 0). This plot illustrates that the rupture of CD44·HA dominates over SAv·biotin over the entire range of experimental loading rates. The probability of the anchor to rupture is though sizeable (between 10 and 30%), and is reduced by approximately two-fold when SAv·biotin is replaced by the more stable TAv·biotin.

Figure 6c compares the numerical calculations for the total rupture probability of the two bonds in series (*i.e.* cumulating the ruptures of either CD44·HA or SAv·biotin) with the expected response for scenarios in which there is only one reversible bond, *i.e.* either CD44·HA or SAv·biotin (for *n* = 1, Eq. (2) simplifies to d*φ*/d*f* = *φk*_off _exp[*fx*_β_/(*k*_B_*T*)]/*r*). From this presentation, it can be appreciated that the curves for CD44·HA alone are very similar to the curves for the two bonds in series, to such an extent that the width and the mean of the distribution are barely affected by the presence of the SAv·biotin bond. Specifically, the decrease in the location of the maxima in the distributions owing to the presence of SAv·biotin remained within ~1 pN. This is below the resolution limit of our experiments, and explains that the occasional breakage of SAv·biotin, when in series with CD44·HA, does not affect the force spectra appreciably that are ultimately used for fitting with the Bell-Evans model (Eq. (1)).

We note that the presentation in Fig. 6c is equivalent to idealized rupture force histograms. The mean rupture forces predicted for CD44·HA alone and SAv·biotin alone are in agreement with the experimental data in Fig. 4d and Supplementary Fig. 8f, respectively. This is to be expected because these data were effectively used as input parameters, and merely confirms that the numerical calculations are correct. Reassuringly, however, the widths of the distributions in Fig. 6c also compare well with the experimental data (*cf.* standard deviations indicated in Fig. 4d and Supplementary Fig. 8f). The width was not an input parameter of the numerical calculations, and the agreement thus provides further support for the validity of the simple theoretical model described by Eq. (2).

Collectively, the experimental results at low and high receptor density demonstrate that the stochastic rupture of individual bonds between HA and CD44 can be reliably characterized in our experimental setup, thus validating that the kinetic parameters shown in Figs 4d and 5c pertain to the genuine interaction of HA with its receptor. The results of the theoretical model are fully consistent with our experimental findings and confirm that the anchors break occasionally but are mostly stable and sufficiently strong not to perturb the analysis of the HA·CD44 interaction with the Bell-Evans model.

## Discussion

In this manuscript we have conceived an experimental platform for the analysis of the nanomechanics of GAG·protein interactions. Focusing on the fundamentally important interaction between HA and its primary cell surface receptor CD44, we have demonstrated how GAG·protein binding can be analyzed at the level of a single GAG chain in a well-defined system that not only preserves the native orientation of the receptor, but which also enables important parameters such as its density and lateral mobility in the membrane to be varied. With this system, the unbinding mechanics of monovalent and multivalent GAG-protein interactions can be measured and directly compared.

HA·CD44 interactions are critical for capturing circulating cells such as activated lymphocytes and neutrophils from the blood flow^5^, enabling their transient adhesion to the luminal surface of the vessel, and the characteristic rolling behavior that precedes extravasation^4^,^7^,^12^,^42^,^43^. Among studies on the interaction of HA with CD44 on a larger scale (involving many HA chains)^7^,^12^,^44^, binding of CD44-positive cells and CD44-coated microspheres to HA-coated surfaces has been reported to strengthen under flow, and it has been proposed that this effect is encoded at the molecular level in the form of an unconventional bond that strengthens under force (catch bond)^7^,^12^. In the measurements described here, the dependence of the mean rupture force on loading rate as well as the magnitude of the standard deviations in mean rupture force (Figs 3e and 4d) are consistent with the predictions of the Bell-Evans model indicating that CD44·HA bond rupture is adequately described by conventional unbinding across a single barrier. These two scenarios, however, are not mutually exclusive: as recently demonstrated by Harder *et al*.^19^ for a different GAG·protein bond, the catch bond behavior may simply not be picked up over the range of loading rates accessible in our experiments. This question clearly deserves further study in the future. On the multi-bond level, the major finding of this study is that multiple CD44·HA bonds along a single HA chain rupture sequentially and independently under load (Fig. 4). Leukocyte rolling along the luminal surface of blood vessels mediated by CD44·HA interactions would thus rely largely on the stochastic rupture and renewal of CD44·HA bonds.

Raman *et al*.^16^ have previously reported single molecule force data on CD44·HA bonds, and it is notable that the mean rupture forces reported with their assay exceed those measured here by about two-fold. The presentation of HA in the earlier work was distinct, with HA polymers being chemically modified at multiple sites along the polymer chain, likely resulting in multiple attachment points to the AFM tip. Furthermore, the CD44 constructs employed and their anchorage to the surface were also different to those in our study. A definitive explanation at this stage is difficult, however, because representative force-separation curves or force histograms were not provided in the earlier study and hence cannot be compared directly with the data presented here.

We have here studied the interaction of HA with a selected construct of CD44. The binding of HA to CD44 on the cell surface in the absence of tensile force has previously been found to be tightly regulated through CD44 post-translational modifications, such as N-glycosylation and terminal sialylation^45^ and receptor clustering. Future studies should focus on the possible contributions of such modifications to the regulation of HA binding under force. In addition, it will also be interesting to compare the mechanical response of CD44·HA bonds with the bonds that HA forms with other cell surface receptors such as the lymphatic vessel endothelial receptor-1 (LYVE1)^46^,^47^,^48^. Such a comparison would be of particular interest, as the lymphatic system experiences lower flow and shear stress than the blood vasculature and how the two receptors respond to the distinct mechanical environments in which they act remains an open question.

Our methodology for protein and GAG immobilization relies on the bio-affinity of selected tags, a feature which offers rapid assembly and precise control on molecular orientations. With its modular design, and the increasing availability of methods for the site-specific tagging of proteins and GAGs^49^ with polyhistidine or biotin, the platform can now be readily applied to a wide range of different proteins and their GAG ligands. Several extensions of the assay platform are conceivable. First, future progress in the conjugation of GAGs may enable the effects of their disposition to be tested through selective tethering *via* the reducing or non-reducing ends as well as permitting analysis of the effects of GAG topology (*e.g.* GAGs containing loops of defined size) on the mechanical response. Second, other force spectroscopy schemes such as force clamps or variations in the direction of applied force could provide further insight into bond mechanics to resolve the presence of multiple unbinding pathways underlying unconventional behavior such as catch bonds^19^, and their effect on multivalent interactions. In this regard, the method should also be applicable to other single-molecule techniques that require immobilization of the interaction partners on surfaces, such as magnetic tweezers, laminar flow assays, acoustic force spectroscopy, or centrifuge force microscopy (see ref. 50 and references therein). Third, other aspects of the *in vivo* conditions also can be readily incorporated to probe the regulatory role of multiple simultaneous protein-GAG interactions. For example, experiments in which GAG-binding proteins are presented in the solution phase jointly with the immobilized receptors would help determine how the modulation of GAG structure by proteins^51^, such as that of HA by TSG-6^52^, affects the nanomechanics of GAG binding to the cell surface. Last but not least, our methodology could conceivably be combined with the analysis of GAG-receptor interactions in live cells. This would enable a direct comparison of responses in biomimetic systems that are well defined and recapitulate selected properties of the cell surface with the more complex (and less well defined) native system.

The ideal anchorage of biomolecules in SMFS is both well defined (in terms of orientation, surface density and lateral mobility) and resistant to tensile force. In practice this represents a trade-off scenario: covalent attachment guarantees high mechanical strength but is often not well defined, whereas attachment through bio-affinity tags is very well defined but the resistance to tensile force is lower. In this regard, the combination of adequately designed control experiments with a simple theoretical model (Eq. (2)) provides an effective tool to assess the viability of non-covalent bonds as anchors in SMFS. Our results demonstrate that interactions of biotin with SAv, and polyhistidine with metal chelators, and the embedding of metal-chelator functionalized lipids in lipid bilayers, are sufficiently strong for the reliable analysis of HA·CD44 interactions. Given the relatively weak nature of typical GAG·protein interactions, it is likely that the anchor stability will also be sufficient to study many other interactions. Where needed, the accessible force range can be extended by the use of TAv instead of SAv. Moreover, the model presented in Eq. (2) may also be used to correct for the effect of anchors in cases where the rupture probability of anchor(s) is comparable to that of GAG·protein interactions, thus further extending the application range. To this end, a data fitting procedure can be envisaged that uses *k*_off_ and *x*_β_ of the anchor bonds (determined with control measurements for each anchor type; *cf.* Fig. S8) as input to extract the *k*_off_ and *x*_β_ values for the GAG·protein interaction from experimental force spectra for the GAG·protein bond in series with the anchor bonds. The sensitivity of the assay to the GAG·protein bond (and thus the accuracy of the correction method) can be expected to be good as long as the rupture probability of the GAG·protein bond is larger than that of the anchors, but to decrease rapidly in the inverse case.

In summary, we have reported the combination of tailored surface functionalization strategies and surface characterization by QCM-D and SE with AFM SMFS to study the nanoscale mechanics of monovalent and multivalent bonds between proteins and GAGs at a level down to a single GAG chain in supramolecular architectures that are designed to reproduce specific aspects of the *in vivo* situation. Applying this approach, we have quantified the mechanical strength of individual CD44·HA bonds and revealed that multiple bonds along a given HA chain rupture sequentially and independently under load. This platform technology should be widely applicable for elucidating molecular mechanisms underlying the response of extracellular matrix and cell surface receptors to mechanical forces.

## Methods

### Buffer, proteins and hyaluronan

A ‘working’ buffer consisting of 10 mM HEPES at pH 7.4 and 150 mM NaCl was used to dilute proteins and HA and throughout all QCM-D and AFM experiments. The full-length ECD (terminating after residue number 267) of human CD44 with a His_10_ tag and a biotin tag at the C-terminus was produced and purified as described in the Supplementary Methods. Monoclonal mouse anti-human CD44 function-blocking antibody BRIC235 (anti-CD44 Ab) was obtained from International Blood Group Reference Laboratory (Bristol, UK). Lyophilized SAv (Sigma Aldrich) was dissolved in ultrapure water to form a stock at 1 mg/ml concentration. TAv was expressed and purified from *E. coli* as described previously^40^, stored at 1 mg/ml in PBS at −20 °C, and further diluted in working buffer for final use. Lyophilized HA polymers with well-defined molecular masses (SelectHA) were purchased from Hyalose (Oklahoma City, OK, USA): HA with a biotin at its reducing end (for AFM SMFS assays) had a molecular mass of 840 ± 60 kDa, and plain HA (for QCM-D assays) had a molecular mass of 250 ± 12 kDa. HA was dissolved, and gently shaken for 2 h in ultrapure water, to provide a stock of 1 mg/ml. Stock solutions of all proteins and HA were aliquoted and stored at −20 °C. Thawed aliquots of proteins were used within a few days, while thawed aliquots of HA were used within a few weeks.

### Lipids

1,2-dioleoyl-sn-glycero-3-phosphocholine (DOPC) was purchased from Avanti Polar Lipids (Alabaster, AL, USA). Tris-NTA-functionalized lipid analogues ((NTA)_3_-SOA)^53^, prepared as previously described^54^ were kindly provided by J. Piehler (Osnabrück University, Germany). Small unilamellar vesicles (SUVs) in working buffer were prepared by sonication from a mixture of DOPC and (NTA)_3_-SOA (95:5 molar ratio), as described previously^55^,^56^. SUVs at a stock concentration of 1 to 2 mg/ml were stored at 4 °C under argon and used within three weeks.

### Substrates

QCM-D sensors with gold coating (QSX301), silica coating (QSX303) and His-tag-capturing coating (QSX340) were obtained from Biolin Scientific (Västra Frölunda, Sweden). Silicon wafers (9 mm × 9 mm) with a native oxide layer of about 2 nm were from University Wafers (South Boston, MA, USA). 100 nm gold coatings were prepared by sputter deposition.

Silica-coated substrates were cleaned with 2% (w/v) SDS for 30 min, rinsed thoroughly with ultrapure water followed by blow-drying with N_2_, and treated with UV/ozone (Bioforce Nanoscience, Ames, IA) for 30 min and stored in air. His-tag-capturing sensors were uses as received and regenerated by 5 mM CuSO_4_. Gold-coated substrates were employed as received and not re-used.

### Surface functionalization

#### Preparation of surfaces for anchorage of biotin-tagged hyaluronan and proteins

Di-end functional oligo(ethylene glycols) (OEGs) were purchased from Polypure (Oslo, Norway), one made of two EG_7_ with hydroxyl groups on one end and connected by a disulfide on the other (OEG disulfide), and the other containing EG_10_ with biotin on one end and a thiol on the other (b-OEG thiol). Gold-coated planar substrates or AFM cantilevers were conditioned by exposure to UV/ozone for 30 min, and then immersed overnight at 4 °C in an ethanolic solution (purity 99.9%; Scharlab S.L.) of OEG disulfide and b-OEG thiol (molar ratio 500:1) at a total concentration of 1 mM. Prior to use, the functionalized substrates were rinsed with ethanol and blow-dried with N_2_. This procedure provides a monolayer of OEG that is inert to non-specific binding of proteins and GAGs; it permits the formation of a monolayer of SAv that serves as a ‘molecular breadboard’ for the controlled anchorage of biotin-tagged molecules^24^,^25^.

#### Preparation of surfaces for anchorage of polyhistidine tagged proteins

His_10_-tagged CD44 was directly immobilized on His-tag-capturing QCM-D sensors. This sensor surface features a passivating layer of poly(ethylene glycol) (PEG) and exposes divalent metal ions for capturing polyhistidine tagged molecules. Alternatively, SLBs containing Ni^2+^-loaded tris-NTA moieties were used to anchor polyhistidine tagged proteins. To this end, silica-coated substrates were conditioned by exposure to UV/ozone for 30 min prior to use, and SLBs were formed from SUVs by the method of vesicle spreading, as described previously^57^.

For anchorage of HA and proteins, the surfaces were incubated with the appropriate molecule (biotin or His tagged) at ambient conditions in working buffer at the required concentration. To obtain receptor monolayers of ‘low’ (~0.07 pmol/cm^2^) and ‘high’ (~1.8 pmol/cm^2^; Supplementary Fig. S3) density, respectively, CD44 was incubated in still solution for 30 min at 0.25 μg/ml and 6.5 μg/ml.

### Quartz crystal microbalance (QCM-D)

QCM-D measures the changes in resonance frequency, ∆*f*, and dissipation, ∆*D*, of a sensor crystal upon molecular adsorption on its surface. The QCM-D response is sensitive to the areal mass density (including hydrodynamically coupled water) and the mechanical properties of the surface-bound layer. To a first approximation, a decrease in frequency (*∆f*) corresponds to increased mass, while a low (high) response in dissipation (*∆D*) corresponds to rigid (soft) films. QCM-D measurements were carried out with a Q-Sense E4 system equipped with Flow Modules (Biolin Scientific AB, Västra Frölunda, Sweden) with flow rates of 5 to 20 μl/min at a working temperature of 23 °C. ∆*f* and ∆*D* were collected at six overtones (*i* = 3, 5, 7, 9, 11, 13). Changes in dissipation, ∆*D*, and normalized frequencies, ∆*f* = ∆*f*_*i*_/*i*, for *i* = 3 are presented. All overtones provided similar information. All experiments were carried out in duplicate; numbers in the manuscript text represent the mean ± variations around the mean.

For dense monolayers of globular proteins, the film thickness was estimated from *d* = −*C*/ρ × Δ*f*, where the density ρ = 1.2 g/cm^3^ represents the protein film density to within an error of less than 20% and *C* = 18.1 ng/cm^2^/Hz the sensor’s mass sensitivity constant^58^.

### Force spectroscopy

AFM experiments were performed on a NanoWizard II system (JPK, Berlin, Germany) in working buffer at ambient conditions, using gold-coated cantilevers with nominal spring constants of 30 or 6 pN/nm (Biolevers), and 60 pN/nm (NPG-10; both from Bruker AFM Probes, USA). The real spring constants were determined by the thermal noise method^59^. Force curves were acquired at selected approach and retract velocities with a maximal applied load of 600 pN and minimal surface dwell time (*i.e*. 0 ms), except otherwise stated. For a given set of AFM probe, surface and interaction settings, several hundreds to thousands of individual force curves were acquired to sample stochastic variations in the interactions. All experiments were performed at least twice with distinct yet identically prepared AFM probes and surfaces.

Force curves were analyzed with JPK data processing software. For quantitative analysis of the stretching of individual HA chains and to extract HA·CD44 bond rupture forces, force-separation curves were fitted to the WLC model^60^. Unless otherwise stated, both persistence length and contour length were adjustable parameters, and only rupture events occurring at tip-sample distances larger than 200 nm were considered, to avoid bias by non-specific tip-sample interactions. Instantaneous loading rates *r* were computed from the effective spring constant *k*_eff_, corresponding to the slope of the WLC best-fit curve close to the rupture (Fig. 3a, inset), and the retract velocity *v* as *r* = *k*_eff_*v*. The kinetic parameters *k*_off_ and *x*_β_ were determined by non-linear regression analysis with OriginPro software (OriginLab, Northampton, MA) of mean rupture force *vs.* instantaneous loading rate data with the Bell-Evans model^13^,^38^, where the standard error of the mean rupture force was considered to compute confidence intervals for the kinetic parameters.

## Additional Information

**How to cite this article**: Bano, F. *et al*. A single molecule assay to probe monovalent and multivalent bonds between hyaluronan and its key leukocyte receptor CD44 under force. *Sci. Rep.* **6**, 34176; doi: 10.1038/srep34176 (2016).

## Supplementary Material

Supplementary Information

## Figures and Tables

**Table 1:** Summary of kinetic parameters obtained from fitting force spectra with the Bell-Evans model.

Anchor 1^a^	Bond probed	Anchor 2^a^	*k*_off_(s^−1^)	*x*_β(nm)_	***cf.*** **Figure**
Cu^2+^ chelate·His_10_	**CD44**·**HA**	b·SAv·b_2_	0.57 ± 0.11	0.60 ± 0.02	4d
SLB·Ni^2+^ chelate·His_10_		b·SAv·b_2_	0.63 ± 0.37	0.67 ± 0.07	5c
Cu^2+^ chelate·His_10_		b·TAv·b_2_	0.44 ± 0.07	0.65 ± 0.02	S7d
b_2_·SAv·b		b·SAv·b_2_	0.43 ± 0.21	0.65 ± 0.06	S9f
b_2_·SAv	**SAv**·**biotin**	thiol-Au	1.40 ± 0.59	0.31 ± 0.03	S8f
b_2_·TAv	**TAv**·**biotin**	thiol-Au	0.85 ± 0.58	0.28 ± 0.04	S8f

^a^All non-covalent interactions in the chain of bonds are listed and indicated by “**•**”; biotin is abbreviated as “b”.
