# Supplementary material for: A single molecule assay to probe monovalent and multivalent bonds between hyaluronan and its key leukocyte receptor CD44 under force
Source: Sci Rep. 2016 Sep 29;6:34176. doi: 10.1038/srep34176 (PMC5040960; doi:10.1038/srep34176)
Supplement: Supplementary Information [file srep34176-s1.pdf]

Supporting Information

A single molecule assay to probe monovalent and multivalent bonds between hyaluronan and its key leukocyte receptor CD44 under force

Fouzia Bano,[1] Suneale Banerji,[2] Mark Howarth,[3] David G. Jackson,[2] Ralf P. Richter[1,4,5,]*

[1]CIC biomaGUNE, Paseo Miramon 182, 20009 Donostia-San Sebastian, Spain; [2]MRC Human Immunology Unit, Weatherall Institute of Molecular Medicine, University of Oxford, Oxford, OX39DS, UK; [3]Department of Biochemistry, University of Oxford, Oxford, OX13QU, UK; [4]Université Grenoble Alpes - CNRS, Laboratoire Interdisciplinaire de Physique (LIPhy), BP 87, 38402 Saint Martin d'Hères, France; [5]University of Leeds, School of Biomedical Sciences and School of Physics and Astronomy, Leeds, LS2 9JT, UK

*Corresponding author, email: rrichter@cicbiomagune.es

SUPPLEMENTARY METHODS

Production of the CD44 ECD with C-terminal biotin and polyhistidine tags:

Preparation of the construct. The ECD from wild type CD44 was amplified from full-length cDNA coding for the standard form of human CD44 using Pfu Ultra AD and the primers hCD44 -14 BamHI F 5' GCGGGATCCGAAGGGGTAGGCACGATGGCCAGG and hCD44 801 10H* XhoI R 5'GCGCCTCGAG*TTA*GTGATGGTGATGGTGATGGTGATGGTGATGCCACTCGATTTTCTGTGCCTCGAAGATGTCATTCAGGCCGGATCTAGGAATTTGGGGTGTCCTTATAGG, where the reverse primer encodes a target site (GLNDIFEAQKIEWH; Avi-tag) for the BirA biotin ligase and a His$_{10}$ tag. Segments of the primers that generate restriction sites are underlined and the stop codon is denoted by italics in the reverse primer. The amplified fragment was cloned into a variant of the pHR Sin vector[1] carrying an internal ribosome entry site (IRES) upstream of a gene encoding emerald GFP. The production of virus-like particles was carried out using HEK 293T cells that were transiently transfected with the pHR Sin based CD44 construct together with pMD.G and p8.91 in 6-well plates using Genejuice (Merck, Darmstadt, Germany) according to the manufacturer's instructions. The HEK 293T cells had been cultured in Dulbecco's modified Eagle's medium (DMEM; Life Technologies) supplemented with 10% fetal calf serum (FCS), 1× penicillin/streptomycin and 1× L-glutamine (both Sigma, supplied at 100×). Supernatant was harvested at 48-72 h post-transfection and passed through a 0.45 μm filter to remove cell debris. Transduction was achieved by adding virus-like particles in 2 ml of the appropriate supernatant to CHO K1 cells that were incubated overnight before the supernatants were replaced with fresh growth medium. After five days, cells with high GFP expression were selected by FACS and stored as a stably transfected cell line for the production of human CD44 ECD Avi His$_{10}$ recombinant protein.

Recombinant protein production and purification. The selected CHO cell line transfected with human CD44 ECD Avi His$_{10}$ was expanded at 37°C, 5% CO_2 in high-glucose DMEM supplemented with 10% FCS, 1 mM sodium pyruvate, 1× penicillin/streptomycin and 1× L-glutamine. On reaching confluence in 15 cm diameter tissue culture petri dishes, the medium was changed for low serum (1% FCS) medium. After a further 4 days, tissue culture supernatant was aspirated and passed over a 0.22 μm filter before being diluted 1:3 in phosphate buffered saline (PBS; First Link Ltd., Wolverhampton, UK). Imidazole (pH 7.4) and NaCl were added to give final concentrations of 20 mM and 300 mM, respectively.

Polyhistidine-tagged protein was extracted by passing the diluted and supplemented supernatant over a 5 ml His Trap column (GE Healthcare) before washing (PBS, 20 mM imidazole, supplemented with a further 150 mM NaCl) and then eluting (PBS, 500 mM imidazole, supplemented with a further 150 mM NaCl). Following elution, the protein was buffer exchanged into PBS and concentrated prior to size exclusion chromatography on a Sephadex 200 10/300GL column (GE Healthcare) that allowed separation of monomer and disulphide-linked dimer species.

Biotin ligation. Recombinant CD44 protein construct was buffer exchanged into 10 mM Tris-HCl, pH 8 and concentrated to 3 mg/ml. Biotin ligation was performed using a BirA-500 kit (Avidity, CO, USA) according to the manufacturer's instructions. Following the reaction, free biotin was removed by size exclusion chromatography.

Additional protein:

Biotinylated complexes of aggrecan G1 domain and cartilage link protein (AG1-LP) were kindly provided by Markku Tammi (University of Eastern Finland, Kuopio, Finland). AG1-LP was purified from bovine articular cartilage and biotinylated as described previously by *N*-hydroxysuccinimide-mediated labeling of surface amines.[2] Lyophilized AG1-LP was dissolved

in working buffer. The complex contains multiple biotin moieties at random (uncontrolled) positions.

Quantification of CD44 surface density by spectroscopic ellipsometry (SE):

To estimate the surface density of CD44, the formation of CD44 monolayers was followed by SE. SE measurements were carried out at room temperature with a M2000V system (J. A. Woollam, Lincoln, NE, USA) and the data were analyzed using CompleteEASE software (J. A. Woollam) following established procedures.[3,4] Briefly, gold-coated silicon wafers functionalized with biotinylated OEG monolayers were installed in a custom-built open cuvette (~180 µl volume, passivated with 10 mg/ml bovine serum albumin for 20 min prior to use). The cuvette featured a magnetic stirrer which was used to homogenize the cuvette content for 20 s after injecting the sample into the cuvette and during rinses with working buffer; sample binding was followed in still solution. In the optical model to fit the data, the opaque gold film and the OEG monolayer were treated as a single isotropic layer and fitted as a B-Spline substrate, and the protein film made of SAv and CD44 was treated as an isotropic and transparent Cauchy layer. Areal protein mass densities were determined through de Feijter's equation,[5] using a refractive index increment of 0.18 cm^3/g.[6,7]

AFM SMFS of SAv•biotin and TAv•biotin interactions:

Gold-coated AFM cantilevers were conditioned by exposure to UV/ozone for 30 min, and then immersed overnight at room temperature in a solution of linear OEG (7 units) with a hydroxyl and a thiol group at the ends (OEG thiol; Polypure) mixed with linear PEG (10 kDa) with a biotin and a thiol group at the ends (b-PEG thiol; IRIS biotech GmbH, Germany) in ultrapure water (total concentration 1 mM, molar ratio 4×10^4 OEG per 1 b-PEG). The substrates were then rinsed in ultrapure water, and immersed in working buffer for their final use.

The mixed OEG/b-PEG monolayer thus formed displayed biotin at a dilution that showed a satisfactory binding frequency in AFM force spectroscopy on planar substrates displaying monolayers of SAv or TAv. Specifically, 65% of all force curves showed no specific unbinding event and 35% showed one event; two or more events were not observed. Monolayers of SAv and TAv were prepared and force data at various retract velocities acquired as described in the Methods section in the main text. Force curves were fit (JPK Data Processing Software) with a freely jointed chain (FJC) model, with the Kuhn segment length fixed to 0.7 nm (which describes the stretching of PEG chains in aqueous solution well over the force range relevant to our assays[8]) and the contour length as the only adjustable parameter. The effective spring constant k_{eff}, corresponding to the slope of the best-fit FJC model curve close to the rupture point, was used to compute the instantaneous loading rate $r = k_{\mathrm{eff}} v$.

SUPPLEMENTARY FIGURES

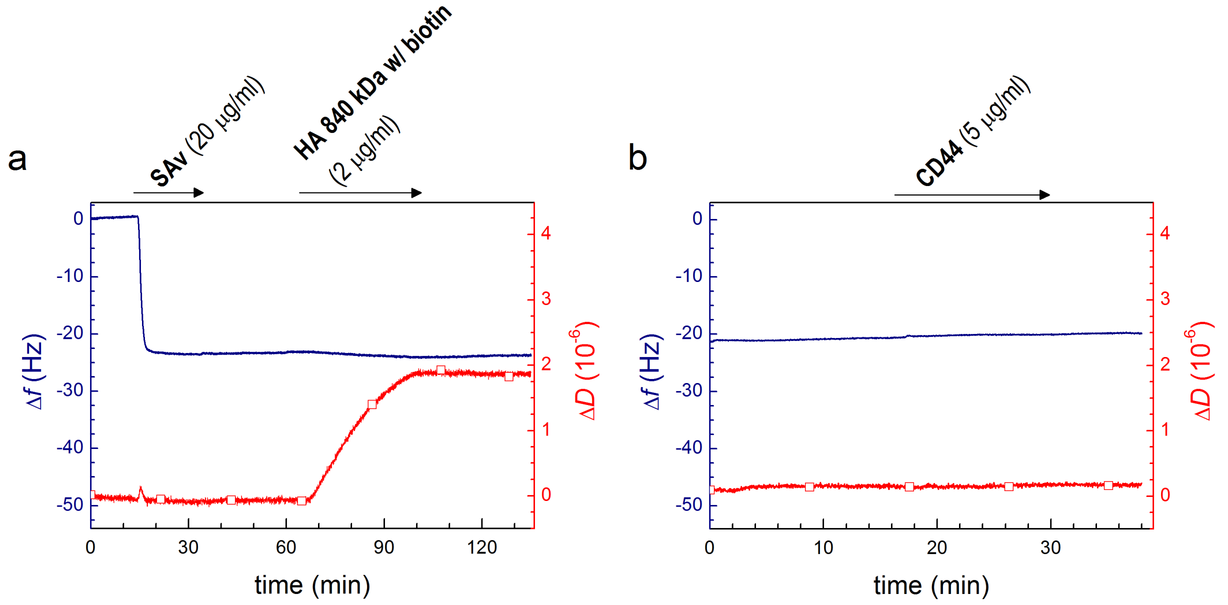

Supplementary Figure S1. QCM-D immobilization assays for end-biotinylated HA (840 kDa, 2.1 µm contour length) on biotinylated OEG monolayers, presented analogous to Fig. 1. The data in (a) confirm formation of a stable and dense SAv monolayer of about 4 nm thickness,[9,10] and subsequent grafting of HA through biotin at the reducing end. The frequency decrease upon HA incubation was small ($\Delta f = -1.3 \pm 0.5$ Hz) and the concomitant dissipation shift large ($\Delta D = 3.1 \pm 0.2 \times 10^{-6}$), consistent with previously reported data, and reflecting the formation of a very soft and hydrated film.[10,11] HA polymers lacking biotin do not bind to SAv monolayers, as shown previously.[10] Data in (b) demonstrate that the CD44 construct employed in this study does not bind to a SAv monolayer that had previously been saturated with soluble biotin. Exposure to biotin does not induce a measurable QCM-D response due to biotin's small size (*cf.* Fig. S3b), and this trace is not shown here.

AFM probes were functionalized with HA as established through the QCM-D assay, but with a reduced HA incubation time of 6 min. At physiological ionic strength, an unperturbed HA chain of 2.1 µm contour length is expected to form a random coil with a radius of gyration of approximately 75 nm.[12] Considering the geometry of the AFM probe and cantilever, and assuming mass-transport limited binding of HA, we can estimate an upper limit of 24 ng/cm^2 for the HA surface coverage, corresponding to a root-mean-square distance between grafting points of 76 nm.[13] This implies that the HA film is in the so-called 'mushroom regime', *i.e.* immobilized polymer coils barely interpenetrate and largely retain their random-coil conformation.[14] Because of the sharpness of the AFM probe (the apex radius is typically 30 nm; *cf.* Fig. 1a) and the grafting density and conformation of HA, we expect only one or at most very few HA molecules to be able to contact the protein-covered surface.

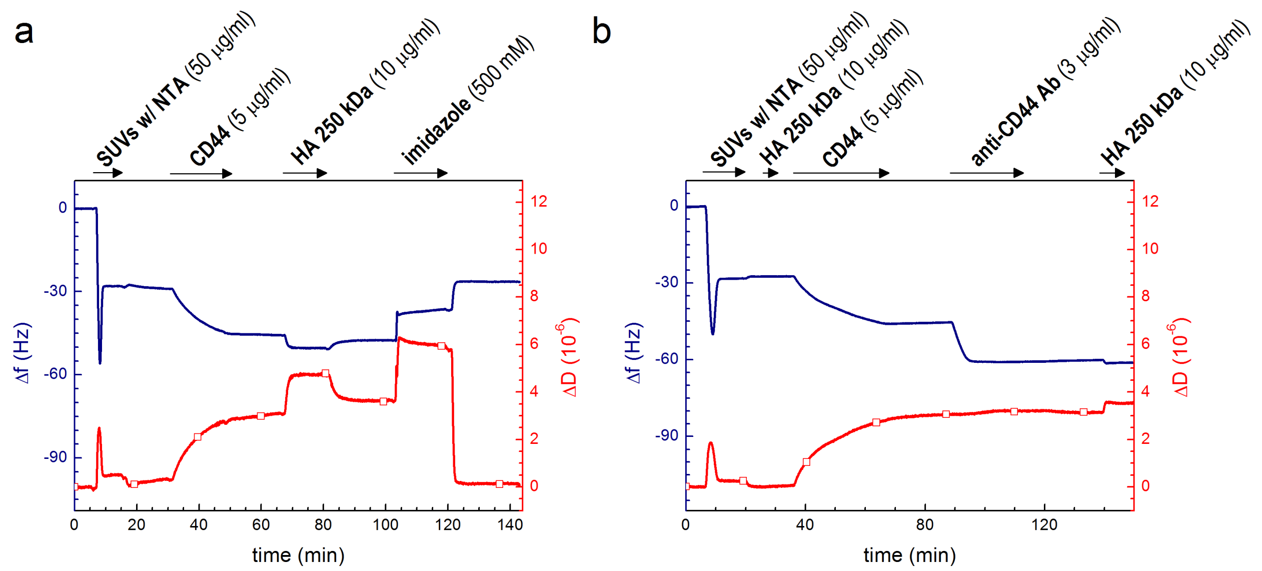

Supplementary Figure S2. QCM-D immobilization assays for CD44 on SLBs, presented analogous to Fig. 1. SLBs were formed by spreading of small unilamellar vesicles (SUVs) composed of a DOPC:$(NTA)_3$-SOA mixture (molar ratio 19:1); the two-phase responses in Δf and ΔD, the small final dissipation shift ($< 0.3 \times 10^{-6}$) and the final frequency shift (-29 ± 1 Hz) are characteristic for the formation of SLBs of good quality.[4,15] QCM-D responses in (a) indicate the formation of a stable and HA-binding CD44 monolayer, and demonstrate that CD44 is specifically immobilized through its polyhistidine tag to the Ni^{2+}-NTA moieties in the SLB (*i.e.* it can be fully eluted in imidazole). Frequency and dissipation shifts at about 120 min reflect the changes in the viscosity and density of the solution due to the presence of imidazole and are not due to surface effects. Data in (b) demonstrate that HA binds through the authentic HA-binding site on CD44 (largely blocked with anti-CD44 Ab).

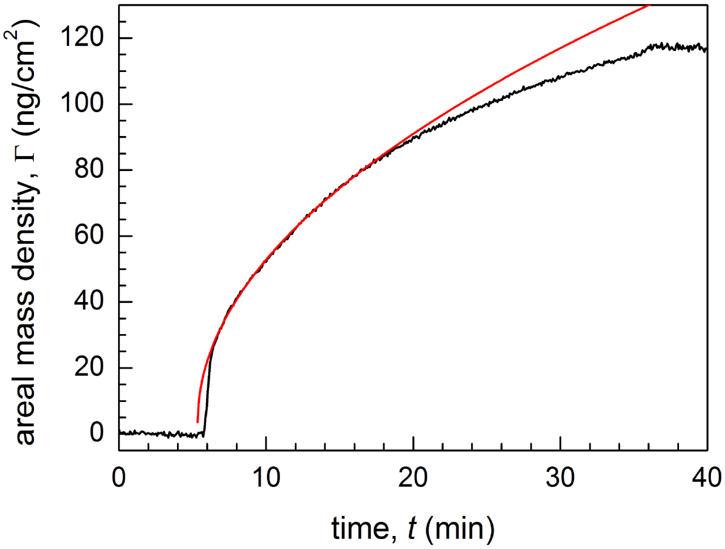

Supplementary Figure S3. Quantification of CD44 receptor density. Experimental data are shown in black, and the red curve is a best-fit theoretical curve for mass-transport limited binding. Immobilization of CD44 *via* its C-terminal biotin tag on a SAv-covered OEG monolayer (Supplementary Fig. S9a-c) was followed by SE to determine the areal protein mass density Γ (see Supplementary Methods for details). The incubation conditions were identical to those used for the preparation of 'high receptor density' samples for AFM force spectroscopy, *i.e.* 6.5 µg/ml CD44 in still solution for 30 min (here from 6 to 36 min). At sufficiently low coverage, binding is expected to be mass-transport limited because the biotin•SAv bond forms fast. For mass-transport limited binding from still solution, binding is expected to scale with the square root of incubation time, with $\Gamma = 2c_\mathrm{b}\sqrt{Dt/\pi}$, where c_b and D are the adsorbate's concentration and diffusion constant in the bulk solution, respectively.[16] This is indeed the case for CD44 up to $\Gamma \approx 80$ ng/cm^2: the red curve corresponds to $\Gamma = \Gamma_0 + A\sqrt{(t - t_0)}$ with $A = 2c_\mathrm{b}\sqrt{D/\pi} = 22.8 \pm 0.2$ ng/(cm^{2}min$^{1/2}$) and where the small offset $\Gamma_0 = 3.5 \pm 0.7$ ng/cm^2 at $t_0 = 5.4 \pm 0.1$ min arises from the brief initial stirring required at the start of incubation for solution homogenization (errors represent confidence intervals from curve fitting). The progressive reduction in binding rate compared to the red curve at $\Gamma > 80$ ng/cm^2 indicates that surface crowding limits binding at high surface coverage.

The surface coverage after 30 min of incubation was 110 ± 5 ng/cm^2 (mean ± variations around the mean from two independent measurements). From SDS PAGE analysis, we estimate the molecular weight of CD44 at 60 kDa, and 'high receptor density' surfaces thus correspond to a surface density of 1.8 pmol/cm^2 and an rms distance of 10 nm. The 'low receptor density' surfaces were obtained at 26-fold lower CD44 concentration (0.25 µg/ml) under otherwise identical incubation conditions. Based on the above equations we predict an areal mass density of 4.2 ng/cm^2 corresponding to a surface density of 0.07 pmol/cm^2 and an rms distance of 50 nm. We expect these estimates to be accurate also for CD44 anchorage *via* polyhistidine tags because binding rates are very similar (compare Fig. 2a with Supplementary Fig. S9a).

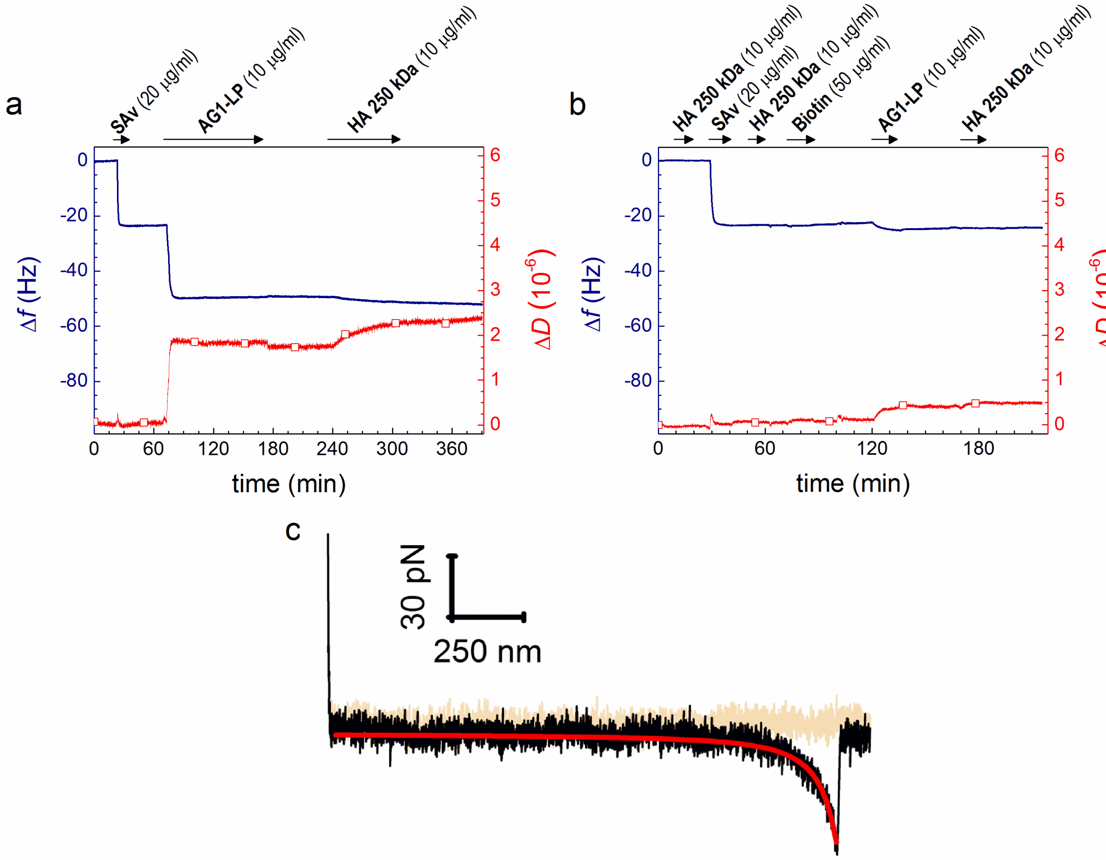

Supplementary Figure S4. Oriented protein immobilization is important for protein functionality. (a-b) QCM-D immobilization assay for a complex of aggrecan G1 domain and cartilage link protein (AG1-LP), presented analogous to Fig. 1. In contrast to CD44 and LYVE-1, AG1-LP was tagged using a procedure that is not site-specific; the tag was biotin and immobilization was performed as for HA (*cf.* Supplementary Fig. S1). Responses upon incubation of AG1-LP indicate stable and specific (*i.e.* largely blockable by saturation of SAv with free biotin) immobilization of the protein through biotin in a monolayer of 5 nm thickness. HA polymer bound stably and specifically to AG1-LP. Compared to CD44 (Fig. 2), the shifts in frequency (-1.7 ± 0.9 Hz) and dissipation ($0.5 \pm 0.2 \times 10^{-6}$) for HA-binding to AG1-LP were small even though AG1-LP is known to bind HA with high affinity.[17-19] The reduced HA binding indicates that only a small fraction of the immobilized AG1-LP is active. Most likely, this is the consequence of tagging AG1-LP at random positions, leading to immobilization with an orientation that renders the HA-binding site of a large fraction of the AG1-LPs inaccessible. (c) Representative force-separation curve (pink – approach, black – retract; approach/retract velocity 1000 nm/s) recorded at maximal AG1-LP surface coverage. The curve shows a single rupture event. The red line is a best-fit WLC model curve with a persistence length of 4.1 nm characteristic for HA (*cf.* Fig. 3). This behavior was observed in 10% of the force curves; 88% showed no rupture event, and 2% showed two or more distinct rupture events. No rupture events (in n = 200 force curves per condition) were observed when either HA on the probe or AG1-LP on the surface were lacking, confirming that the observed interactions are specific. The frequency of binding events is remarkably small, if one considers that the employed HA polymers are long enough to bind about 100 AG1-LPs simultaneously, and that the polymer coil can readily explore a surface area containing tens of AG1-LPs. It is consistent with the limited activity of immobilized AG1-LP observed by QCM-D, and illustrates the importance of ensuring precise protein orientation.

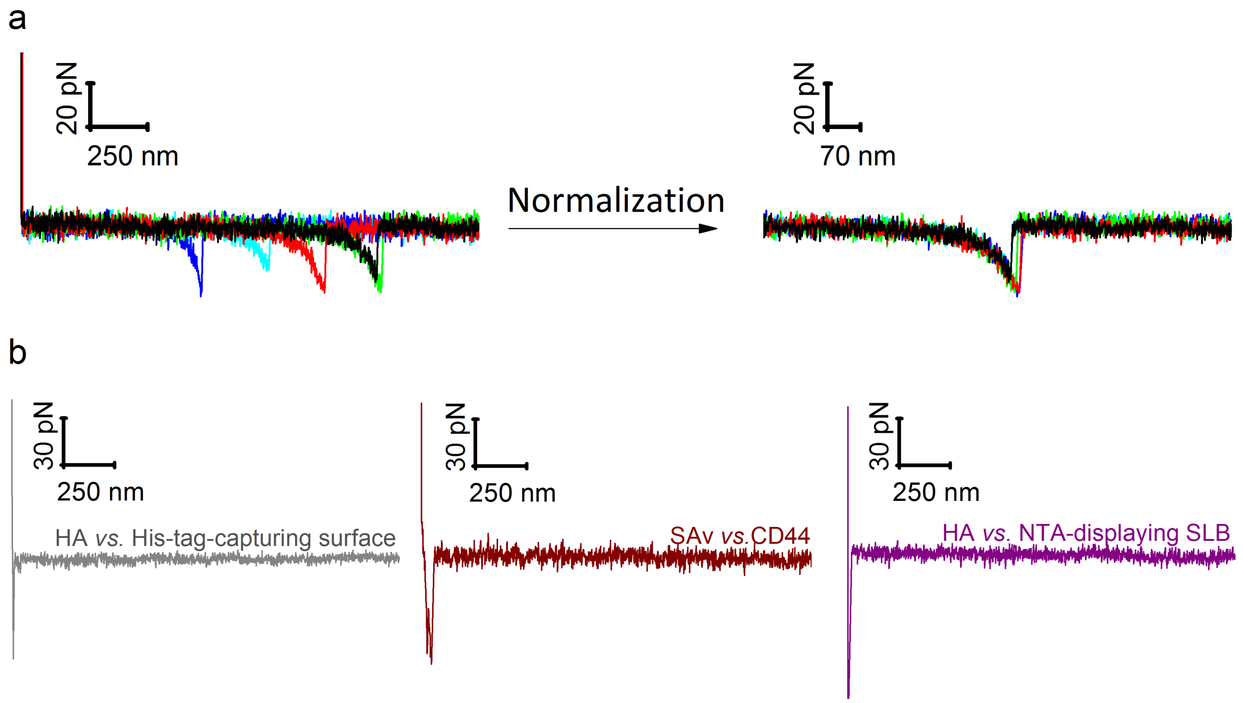

Supplementary Figure S5. Supplementary force spectroscopy data for HA•CD44 interactions. (a) Set of 5 randomly selected force-separation curves (retract velocity 1000 nm/s; conditions as in Fig. 3) featuring a single specific rupture event at various separation distances. The extension curves overlap when normalized,[20] confirming that interactions between a single HA chain and CD44 are probed. (b) Selected force-separation curves, registered at a retract velocity of 2000 nm/s, for control conditions as indicated. No specific rupture events are observed, and this feature is representative for all force curves acquired for the control conditions (*n* = 200 each).

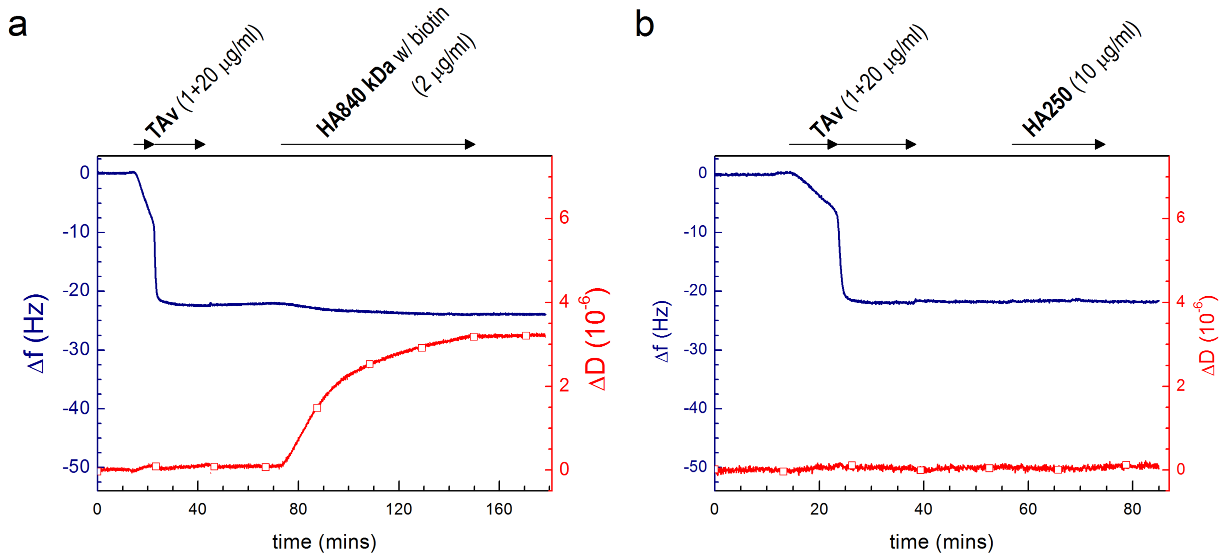

Supplementary Figure S6. QCM-D immobilization assays for biotinylated HA on TAv monolayers, presented analogous to Fig. 1. The QCM-D responses after TAv binding, and upon subsequent incubations with biotinylated HA (a) are virtually identical to those observed for SAv (*cf.* Supplementary Fig. S1a), confirming that the switch from SAv to TAv does not alter the surface density and organization of HA. The lack of binding for biotin-free HA (b) confirms specific immobilization through biotin. TAv was incubated sequentially at two distinct concentrations (first at 1 µg/mL and then at 20 µg/mL) for technical reasons; this does not affect the properties of the final TAv monolayer.

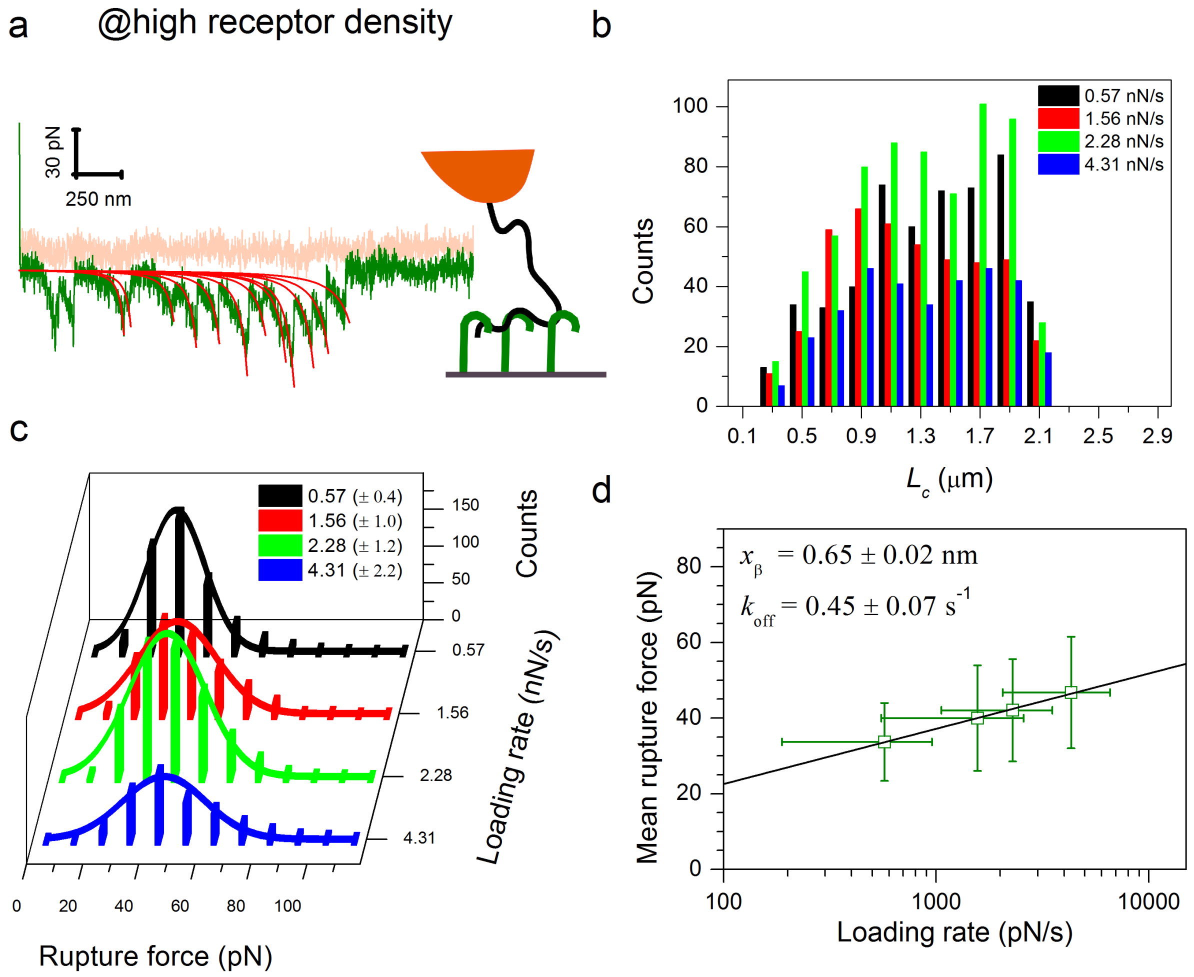

Supplementary Figure S7. Immobilization of HA through TAv instead of SAv does not affect the CD44•HA force spectroscopy data. Force spectroscopy data shown here were obtained with HA immobilized *via* TAv and high CD44 surface density (rms inter-CD44-distance ~10 nm) on a His-tag-capturing sensor. (a) Representative force-separation curve (pink – approach, green – retract; approach/retract velocity 1000 nm/s). The red lines are best-fit WLC model curves (L_p = 4.1 nm fixed). Histograms of effective contour lengths (b) and rupture forces (c), and dynamic force spectra (d) are displayed analogous to Fig. 3c-e. Mean rupture forces and standard deviations are virtually identical to those obtained with HA immobilized *via* SAv (*cf.* Fig. 4d and Table 1).

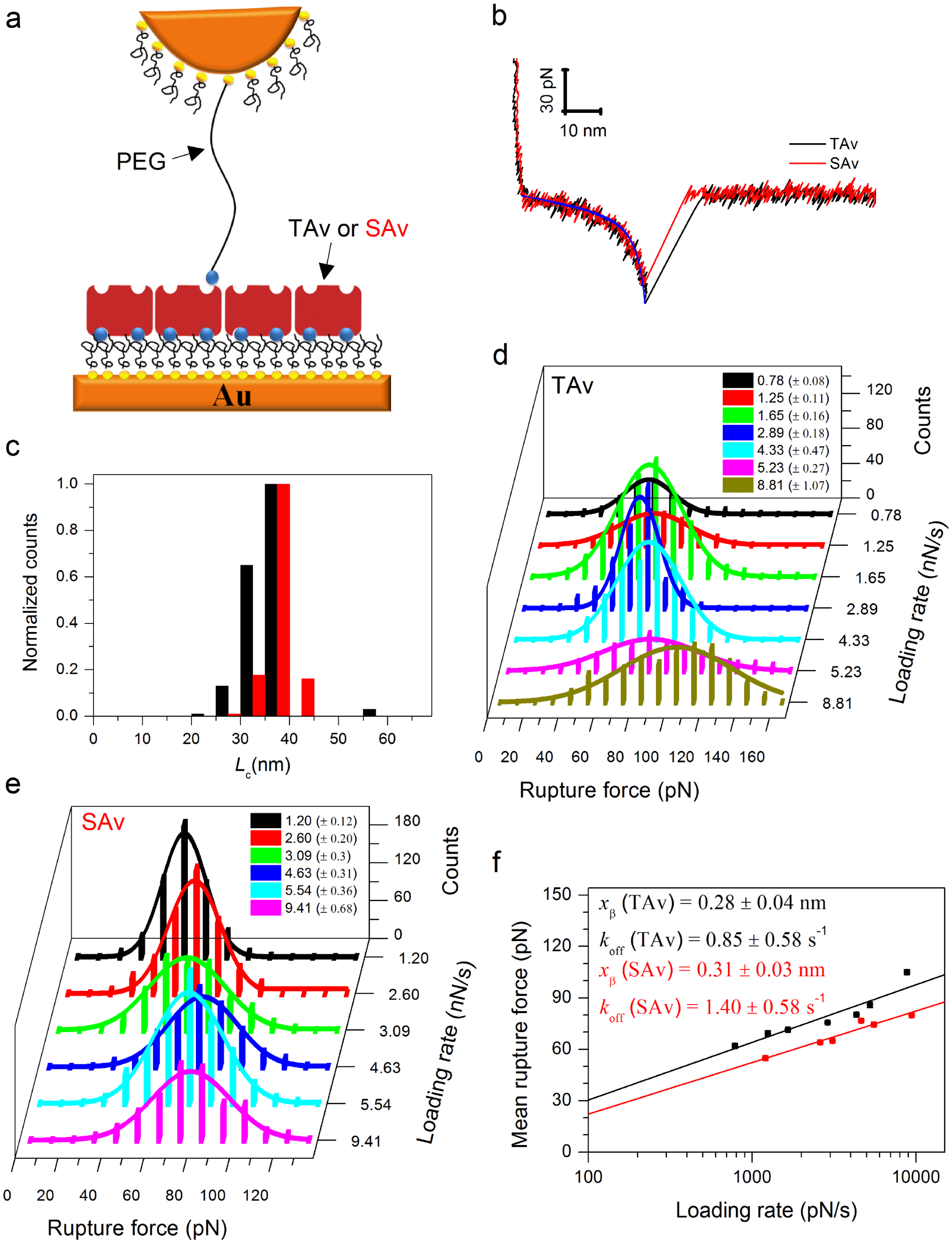

Supplementary Figure S8. Biotin unbinds at larger forces from TAv than from SAv. (a) Schematic representation of the AFM SMFS assay (not to scale): biotin is attached through a PEG-thiol linker to a gold-coated AFM tip, and monolayers of TAv and SAv were formed as shown in Supplementary Figs. S6 and S1, respectively. (b) Representative force-separation curves (black – TAv, red – SAv; retract velocity 300 nm/s). The blue line is a best-fit FJC model curve (see Supplementary Methods for details). (c) Histograms of the effective contour length L_c of the b-PEG chain (black – TAv, red – SAv; retract velocity 300 nm/s). Rupture force histograms for TAv (d) and SAv (e) are displayed analogous to Fig. 3d. (f) Dynamic force spectra (black – TAv, red - SAv) obtained from the data in (d) and (e) and displayed analogous to Fig. 3e except for the error bars which here represent s.e.m, and these are essentially smaller than the symbol size. The data are in good agreement with previous reports by Chivers et al. (TAv•biotin: x_β = 0.26 ± 0.01 nm, k_{off} = 0.51 ± 0.04 s^{-1}; SAv•biotin: x_β = 0.28 ± 0.02 nm, k_{off} = 0.88 ± 0.16 s^{-1}).[21]

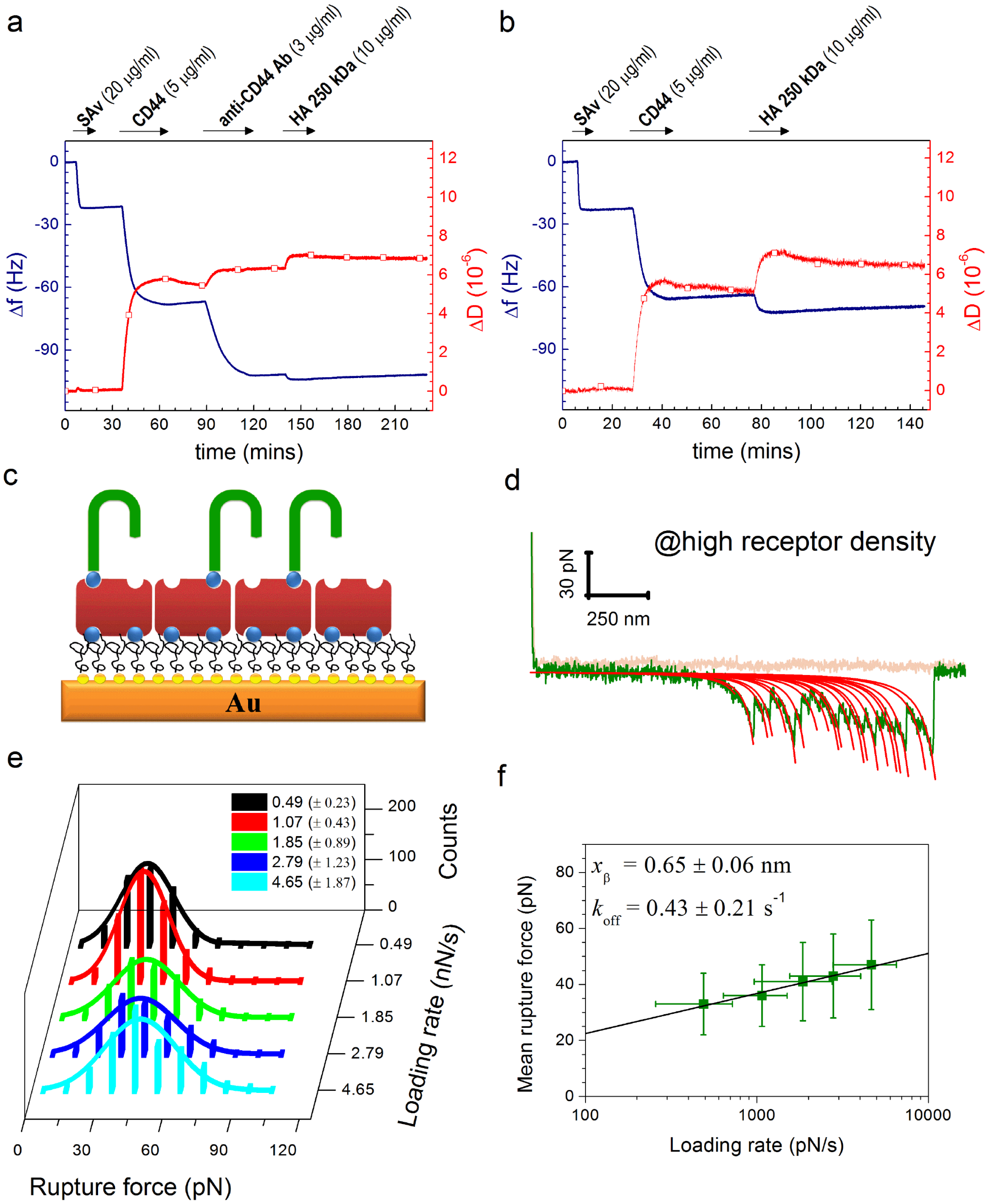

Supplementary Figure S9. Immobilization of CD44 through the biotin tag instead of the polyhistidine tag (both located at the C-terminus) does not affect the force spectroscopy data. (a-b) QCM-D immobilization assays, presented analogous to Fig. 1. QCM-D responses in (a) indicate formation of a stable and HA-binding CD44 monolayer on SAv (see Supplementary Fig. S1b for a demonstration that binding is specific through biotin). Data in (b) demonstrate that HA binds through the authentic HA-binding site on CD44 (largely blocked with anti-CD44 Ab). (c) Representation of the surface architecture (not to scale) displaying CD44 anchored analogous to HA (*cf.* Fig. 1 and Supplementary Fig. S1) on a SAv-coated biotinylated OEG monolayer. (d-f) Force spectroscopy data obtained at high CD44 surface density (CD44 was incubated at 6.5 µg/ml for 30 min). (d) A representative force-separation curve (pink – approach, green – retract; retract velocity 2000 nm/s). The red lines are best-fit WLC model curves (L_p = 4.1 nm fixed). (e) Rupture force histograms displayed analogous to Fig. 3d. (f) Dynamic force spectra displayed analogous to Fig. 3e. Mean rupture forces and standard deviations are virtually identical to those obtained with CD44 immobilized through the polyhistidine tag (*cf.* Fig. 4d and Table 1).

SUPPORTING REFERENCES

1 Naldini, L., Blomer, U., Gallay, P., Ory, D., Mulligan, R., Gage, F. H., Verma, I. M. & Trono, D. In vivo gene delivery and stable transduction of nondividing cells by a lentiviral vector. *Science* **272**, 263-267, (1996).

2 Tammi, R., Agren, U. M., Tuhkanen, A. L. & Tammi, M. Hyaluronan metabolism in skin. *Prog. Histochem. Cytochem.* **29**, 1-81, (1994).

3 Dubacheva, G. V., Curk, T., Mognetti, B. M., Auzely-Velty, R., Frenkel, D. & Richter, R. P. Superselective targeting using multivalent polymers. *J Am Chem Soc* **136**, 1722-1725, (2014).

4 Eisele, N. B., Frey, S., Piehler, J., Görlich, D. & Richter, R. P. Ultrathin nucleoporin phenylalanine–glycine repeat films and their interaction with nuclear transport receptors. *EMBO Rep.* **11**, 366-372, (2010).

5 De Feijter, J. A., Benjamins, J. & Veer, F. A. Ellipsometry as a tool to study the adsorption behavior of synthetic and biopolymers at the air–water interface. *Biopolymers* **17**, 1759-1772, (1978).

6 Richter, R. P., Rodenhausen, K. B., Eisele, N. B. & Schubert, M. in *Ellipsometry of Functional Organic Surfaces and Films* (eds Hinrichs, K. *et al.*) 223-248 (Springer 2014).

7 Zhao, H., Brown, P. H. & Schuck, P. On the distribution of protein refractive index increments. *Biophys J* **100**, 2309-2317, (2011).

8 Oesterhelt, F., Rief, M. & Gaub, H. E. Single molecule force spectroscopy by AFM indicates helical structure of poly(ethylene-glycol) in water. *New J. Phys.* **1**, 6, (1999).

9 Migliorini, E., Thakar, D., Sadir, R., Pleiner, T., Baleux, F., Lortat-Jacob, H., Coche-Guerente, L. & Richter, R. P. Well-defined biomimetic surfaces to characterize glycosaminoglycan-mediated interactions on the molecular, supramolecular and cellular levels. *Biomaterials* **35**, 8903-8915, (2014).

10 Baranova, N. S., Nileback, E., Haller, F. M., Briggs, D. C., Svedhem, S., Day, A. J. & Richter, R. P. The inflammation-associated protein TSG-6 cross-links hyaluronan via hyaluronan-induced TSG-6 oligomers. *J. Biol. Chem.* **286**, 25675-25686, (2011).

11 Richter, R. P., Hock, K. K., Burkhartsmeyer, J., Boehm, H., Bingen, P., Wang, G., Steinmetz, N. F., Evans, D. J. & Spatz, J. P. Membrane-grafted hyaluronan films: a well-defined model system of glycoconjugate cell coats. *J. Am. Chem. Soc.* **129**, 5306-5307, (2007).

12 Takahashi, R., Al-Assaf, S., Williams, P. A., Kubota, K., Okamoto, A. & Nishinari, K. Asymmetrical-flow field-flow fractionation with on-line multiangle light scattering detection. 1. Application to wormlike chain analysis of weakly stiff polymer chains. *Biomacromolecules* **4**, 404-409, (2003).

13 Goldstein, B., Coombs, D., He, X., Pineda, A. R. & Wofsy, C. The influence of transport on the kinetics of binding to surface receptors: application to cells and BIAcore. *J Mol Recognit* **12**, 293-299, (1999).

14 de Gennes, P. G. Polymers at an interface; a simplified view. *Adv. Colloid Interface Sci.* **27**, 189-209, (1987).

15 Richter, R. P., Berat, R. & Brisson, A. R. Formation of solid-supported lipid bilayers: an integrated view. *Langmuir* **22**, 3497-3505, (2006).

16 Hermens, W. T., Benes, M., Richter, R. & Speijer, H. Effects of flow on solute exchange between fluids and supported biosurfaces. *Biotechnol. Appl. Biochem.* **39**, 277-284, (2004).

17 Christner, J. E., Brown, M. L. & Dziewiatkowski, D. D. Affinity binding of the cartilage proteoglycan protein-keratan sulfate core to immobilized hyaluronic acid. *Anal Biochem* **90**, 22-32, (1978).

18 Tengblad, A. A comparative study of the binding of cartilage link protein and the hyaluronate-binding region of the cartilage proteoglycan to hyaluronate-substituted Sepharose gel. *Biochem. J.* **199**, 297-305, (1981).

19 Nieduszynski, I. A., Sheehan, J. K., Phelps, C. F., Hardingham, T. E. & Muir, H. Equilibrium-binding studies of pig laryngeal cartilage proteoglycans with hyaluronate oligosaccharide fractions. *Biochem. J.* **185**, 107-114, (1980).

20 Janshoff, A., Neitzert, M., Oberdorfer, Y. & Fuchs, H. Force Spectroscopy of Molecular Systems-Single Molecule Spectroscopy of Polymers and Biomolecules. *Angew. Chem. Int. Ed. Engl.* **39**, 3212-3237, (2000).

21 Chivers, C. E., Crozat, E., Chu, C., Moy, V. T., Sherratt, D. J. & Howarth, M. A streptavidin variant with slower biotin dissociation and increased mechanostability. *Nat. Methods* **7**, 391-393, (2010).
